# Regulation of the apical extension morphogenesis tunes the mechanosensory response of microvilliated neurons

**DOI:** 10.1371/journal.pbio.3000235

**Published:** 2019-04-19

**Authors:** Laura Desban, Andrew Prendergast, Julian Roussel, Marion Rosello, David Geny, Claire Wyart, Pierre-Luc Bardet

**Affiliations:** 1 Institut du Cerveau et de la Moelle épinière (ICM), Inserm U 1127, CNRS UMR 7225, Sorbonne Université, Paris, France; 2 Institut Curie, PSL Research University, INSERM U934, CNRS UMR3215, Paris, France; 3 Plateforme d’imagerie NeurImag de l’Institut de Psychiatrie et Neurosciences de Paris (IPNP), Inserm U 1266, Paris, France; Baylor College of Medicine, UNITED STATES

## Abstract

Multiple types of microvilliated sensory cells exhibit an apical extension thought to be instrumental in the detection of sensory cues. The investigation of the mechanisms underlying morphogenesis of sensory apparatus is critical to understand the biology of sensation. Most of what we currently know comes from the study of the hair bundle of the inner ear sensory cells, but morphogenesis and function of other sensory microvilliated apical extensions remain poorly understood. We focused on spinal sensory neurons that contact the cerebrospinal fluid (CSF) through the projection of a microvilliated apical process in the central canal, referred to as cerebrospinal fluid-contacting neurons (CSF-cNs). CSF-cNs respond to pH and osmolarity changes as well as mechanical stimuli associated with changes of flow and tail bending. In vivo time-lapse imaging in zebrafish embryos revealed that CSF-cNs are atypical neurons that do not lose their apical attachment and form a ring of actin at the apical junctional complexes (AJCs) that they retain during differentiation. We show that the actin-based protrusions constituting the microvilliated apical extension arise and elongate from this ring of actin, and we identify candidate molecular factors underlying every step of CSF-cN morphogenesis. We demonstrate that Crumbs 1 (Crb1), Myosin 3b (Myo3b), and Espin orchestrate the morphogenesis of CSF-cN apical extension. Using calcium imaging in *crb1* and *espin* mutants, we further show that the size of the apical extension modulates the amplitude of CSF-cN sensory response to bending of the spinal cord. Based on our results, we propose that the apical actin ring could be a common site of initiation of actin-based protrusions in microvilliated sensory cells. Furthermore, our work provides a set of actors underlying actin-based protrusion elongation shared by different sensory cell types and highlights the critical role of the apical extension shape in sensory detection.

## Abstract 2

A study of differentiating spinal sensory neurons in vivo uncovers critical factors required for the morphogenesis of sensory microvilli and reveals fine modulation of mechanosensory responses by microvillus length.

## Introduction

Cells assume a variety of shapes known to be instrumental in their specific functions. Morphologies of neurons are among the most complex and diverse: they extend protrusions, build dendritic arborizations, and project axons in an intricate and stereotypical manner [1]. Sensory neurons, in particular, display a high degree of specialization, allowing them to detect sensory cues, transduce the information into electrical signals, and propagate action potentials to the central nervous system. Much effort has been invested in the identification of molecular determinants underlying sensory detection and transduction, mostly in hair cells—non-neuronal sensory cells—to decipher the pathways and morphological features of audition [2,3]. In contrast, mechanisms responsible for the morphological differentiation of sensory neurons and the role of their morphology in sensory functions remain elusive. Here, we tackle these questions by studying spinal cerebrospinal fluid-contacting neurons (CSF-cNs), a class of evolutionarily conserved sensory neurons [4]. We investigated the molecular mechanisms underlying morphogenesis of the CSF-cN apical extension and its role in sensory-mediated activity.

Spinal CSF-cNs are located along the central canal, into which they extend an apical extension composed of 1 motile cilium surrounded by actin-based protrusions that are in direct contact with the CSF [5–8]. Over the past decade, multiple studies investigated the hypothesis of Kolmer and Agduhr [9,10] that CSF-cNs are sensory and detect cues via their apical extension reminiscent of the hair bundle in hair cells. Diverse sensory cues can be detected by CSF-cNs, including changes in pH and osmolarity [11,12] and mechanical stimuli associated with CSF flow [11,13] or spine curvature [14]. CSF-cNs express the transient potential receptor channel 3 (Trpp3), further referred to as Pkd2l1 (for Polycystic kidney disease-2-like 1)[8,12,15]. While CSF-cN response to pH changes seems to be carried by Acid-Sensing Ion Channels (ASICs) rather than by Pkd2l1 [11,12], multiple lines of evidence indicate that Pkd2l1 is necessary for responses to mechanosensory inputs, such as changes of osmolarity, CSF flow, and tail bending [12–14]. The Pkd2l1 channel is abundantly enriched at the level of CSF-cN microvilli [13], reinforcing previous suggestions that the CSF-cN apical extension might play a similar role in sensory functions to the hair bundle in hair cells [16]. During embryogenesis, spinal CSF-cNs are born from 2 ventral progenitor domains in mouse and zebrafish [17–19]. This dual origin is associated with 2 functionally and morphologically distinct subpopulations organized in 2 ventral and dorsolateral rows [13,20,21]. We previously showed that ventral and dorsolateral CSF-cNs exhibit differently shaped apical extensions [21]. When considered with the localization of Pkd2l1 at the microvilli [13], this observation suggests that such morphological distinction might underlie distinct sensory functions described in the 2 CSF-cN subtypes. How CSF-cNs form their apical extension and how their shape contributes to sensory function remain unknown.

The CSF-cN neural progenitors are epithelial cells displaying an apicobasal polarity with their apical side facing the CSF. At this side, CSF-cN precursors form apical junctional complexes (AJCs), including adherens junctions and tight junctions, closely associated with actin filaments organized in a circumferential belt [22]. Together, AJCs and actin cytoskeleton control the cohesiveness and permeability of the developing tissue [23,24]. The apical localization and maintenance of AJCs rely on the activity of a family of transmembrane proteins, the Crumbs family [25]. Neural progenitors give birth to differentiating neurons through self-renewing asymmetric divisions [26]. It was recently shown that the daughter cell fated to become a neuron loses its apical domain and adhesiveness [27], a process thought to be key for its subsequent neuronal differentiation [23,24]. During their differentiation, many neurons then exhibit actin-based protrusions in a polarized fashion, which indicates that they re-establish an apicobasal polarity. It is unknown whether CSF-cNs also differentiate through a process of apical domain loss and polarity re-establishment or not.

In the case of sensory neurons, specialized protrusions called microvilli can arise at their apical side to constitute the sensory organelle. Our current understanding of the formation of actin-based protrusions comes from studies of filopodia—short-lived thin processes—and involves 2 critical steps: initiation and elongation [28,29]. The initiation of filopodia is characterized by the recruitment of actin nucleators and organizers to the apical membrane specifically. Insulin Receptor Substrate protein of 53 kDa (IRSp53) family members, including Brain-specific angiogenesis inhibitor 1-associated protein (Baiap) and Baiap-like proteins, are key elements of this step because they are capable of inducing membrane curvature and recruiting actin nucleators essential for the initiation of protrusions [30]. So far, it is not known whether this model translates to long-lived microvilli found in sensory cells [31].

After their initiation, membrane protrusions lengthen and, in the case of microvilli, stabilize. The elongation of sensory microvilli has been thoroughly studied in hair cells of the inner ear that form rigid microvilli derivatives referred to as stereocilia [31,32]. Through the isolation of mutations responsible for deafness in humans and animal models, multiple genes have been linked to morphogenesis of the sophisticated hair cell’s sensory organelle [33]. Among these genes, *espin* and *myo3b* encode proteins that have been shown to interact and ensure the proper morphogenesis of stereocilia [34–36]. Loss of Espin function was further demonstrated to lead to stereocilia defects and, eventually, degeneration followed by hair cell death [33,37]. It is not clear whether these interaction and morphogenetic mechanisms translate to other microvilliated sensory cells.

Here, we monitor CSF-cN differentiation using long-term time-lapse imaging of F-actin and report 3 critical steps in their morphogenesis: (i) the presence of a ring of actin at the level of the AJCs, (ii) the initiation, and (iii) the elongation of actin-based protrusions from this actin ring. We show that, in contrast to other newborn neurons, CSF-cNs do not withdraw from the ependymal surface to migrate and differentiate. Instead, they appear to retain their apical cell polarity throughout differentiation, an original element of their morphogenesis. We describe new molecular factors known to be involved in cell polarity and/or morphogenesis, which are specifically enriched in CSF-cNs in the spinal cord. We identified a polarity factor—*crb1*—and several actin cytoskeleton interactors—*baiap2a*, *baiap2l1b*, *myo3b*, and *espin*. Our observations suggest a model in which these factors cooperate to regulate each step of the formation of the CSF-cN apical extension. Investigating the phenotype of a newly generated *crb1* mutant, we describe a critical role of Crumbs 1 in the morphogenesis of CSF-cN apical extensions. Using a dominant-negative (DN) form of Myosin 3b, we demonstrate that this protein is required to address the actin-bundling factor Espin to the apical extension and enable it to reach the proper size. The analysis of a newly generated *espin* mutant indicates that Espin actin-bundling activity is necessary for the correct elongation of the microvilli constituting the CSF-cN apical extensions. Finally, functional analysis demonstrated that shorter apical extensions were associated to reduced mechanosensory responses in CSF-cNs lacking Crb1 or Espin. All together, these results provide insightful elements to build a mechanistic model of the formation of the apical extension by CSF-cNs and how its structure contributes to CSF-cN sensory function.

## Results

### Critical steps to form a specialized apical extension

**Fig 1:**
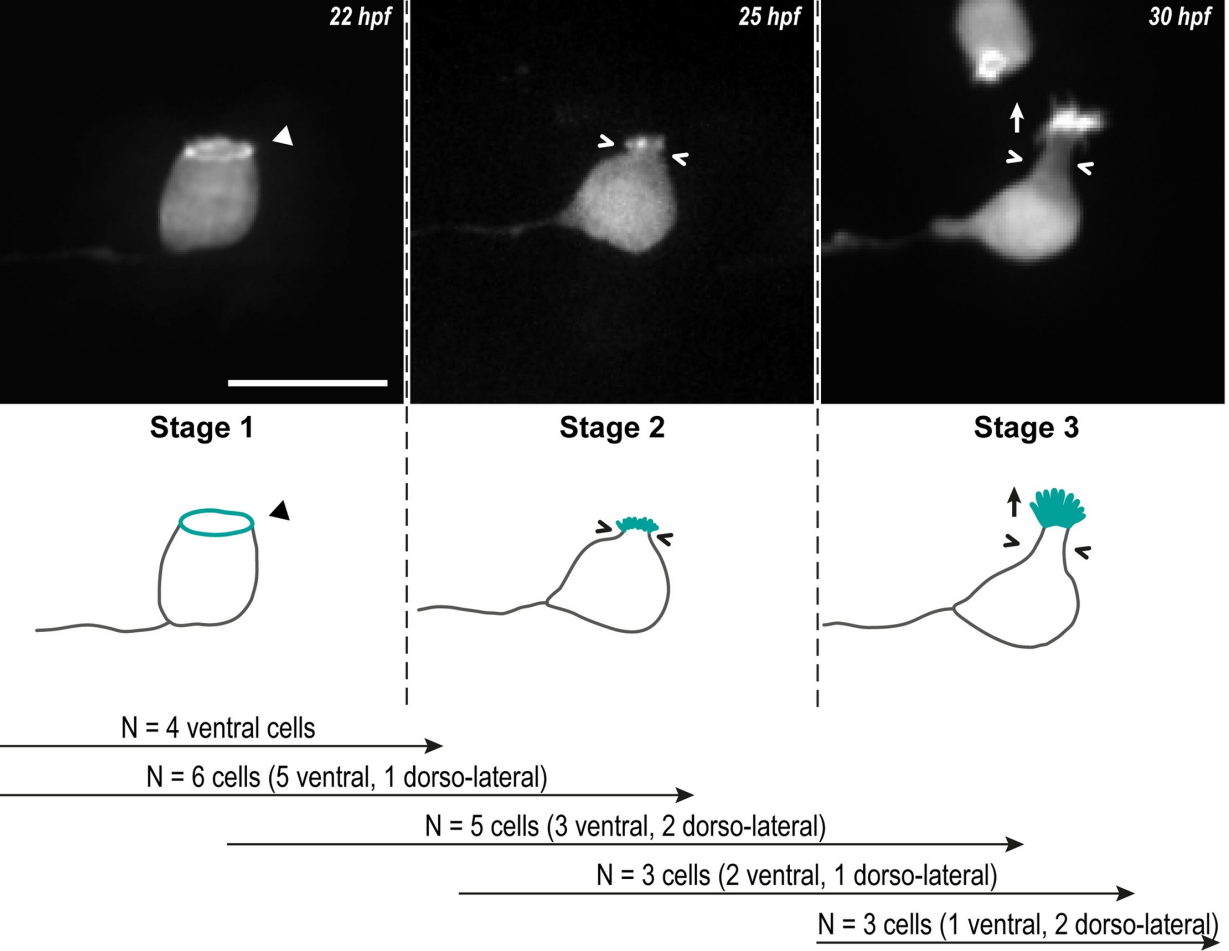
CSF-cNs go through 3 critical steps to form their apical extension. Z-projections from time-lapse acquisitions (top panels; lateral views of ventral CSF-cNs with rostral to the left) and schematics (bottom panels) showing the 3 stages CSF-cNs go through during the formation of their AE. The CSF-cN soma becomes round and short actin-based protrusions (Stage 2) arise from the ring of actin (Stage 1, arrowhead) concomitantly with a subapical constriction (Stages 2 and 3; chevrons). Gradually, protrusions lengthen to form the AE (Stage 3; arrow) characteristic of differentiated CSF-cNs. Data collected over 13 time-lapse sessions for 15 ventral and 6 dorsolateral cells. The number of cells imaged transitioning from one stage to the next is indicated in the bottom legend. Scale bar, 10 μm. AE, apical extension; CSF-cN, cerebrospinal fluid-contacting neuron. hpf, hours post fertilization.

**Fig 2:**
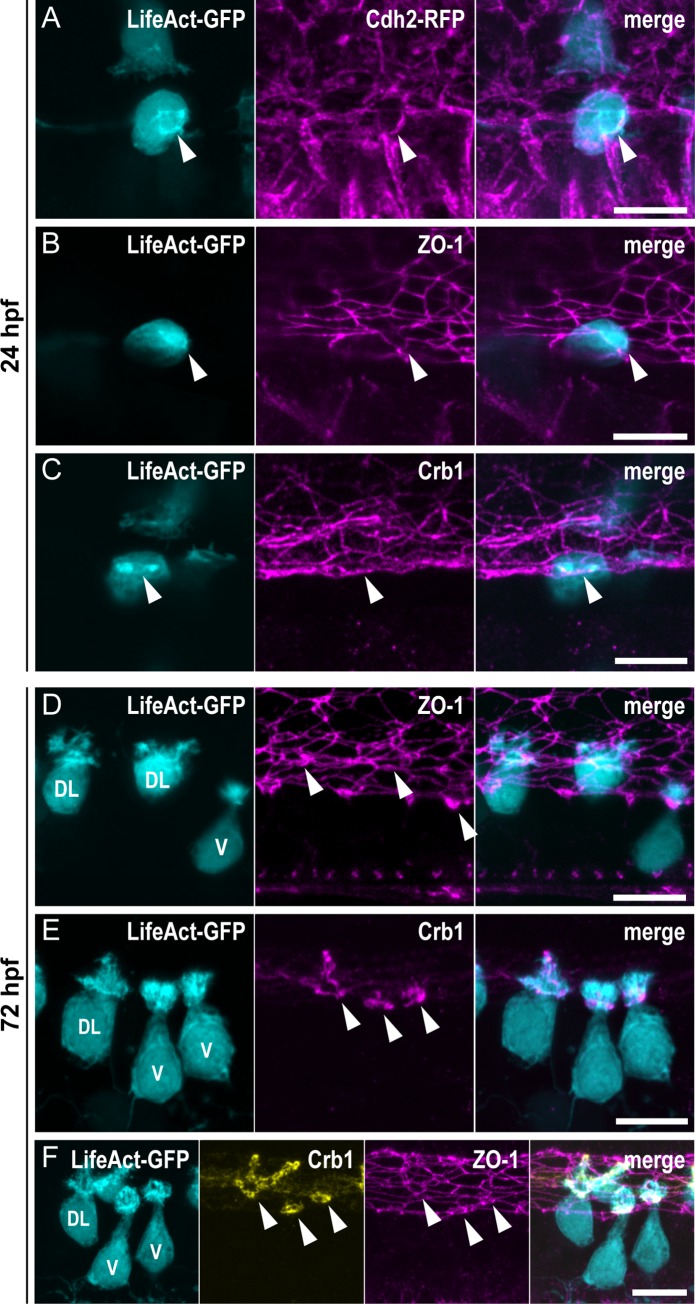
The ring of actin colocalizes with the CSF-cN apical junctional complexes. (A–C) Z-projections from lateral views of the spinal cord in 24-hpf embryos showing the colocalization of the ring of actin (LifeAct; arrowheads) with different markers of the AJCs: (A) Cdh2 for adherens junctions, (B) ZO-1 for tight junctions, and (C) Crb1 for the apical domain. (A) Double immunostaining for GFP and RFP in triple transgenic *Tg(pkd2l1*:*Gal4*,*UAS*:*LifeAct-GFP;cryaa*:*V*,*cdh2*:*cdh2-RFP)* embryos. (B, C) Double immunostaining for GFP and (B) ZO-1, or (C) Crb1 in *Tg(pkd2l1*:*Gal4*,*UAS*:*LifeAct-GFP;cryaa*:*V)* embryos. (D–F) Z-projections from lateral views of 72-hpf *Tg(pkd2l1*:*Gal4*,*UAS*:*LifeAct-GFP;cryaa*:*V)* larvae immunostained for GFP and (D) ZO-1, (E) Crb1, or (F) ZO-1 and Crb1. The markers ZO-1 and Crb1 are retained at the AJCs (arrowheads) in both V and DL CSF-cNs after differentiation. In DL cells, Crb1 expands to the apical extension. Scale bars, 10 μm. AJC, apical junctional complexes; Cdh2, Cadherin 2; Crb1, Crumbs 1; CSF-cN, cerebrospinal fluid-contacting neurons; DL, dorso-lateral; GFP, green fluorescent protein; hpf, hours post fertilization; RFP, red fluorescent protein; V, ventral; ZO-1, zonula-occludens-1.

To investigate the formation of the actin-based apical extension in CSF-cNs, we used the mosaic expression of LifeAct, a marker of F-actin [38], under the control of the *pkd2l1* promoter [39]. We performed long-term time-lapse imaging on single CSF-cNs in live zebrafish from 20 to 22 hours post-fertilization (hpf) (S1 Movie). CSF-cNs first appear as cuboidal, with a growing axon and a ring of actin at the apical side (Fig 1, Stage 1). Then, while the CSF-cN soma becomes round, short protrusions arise from the actin ring (Fig 1, Stage 2). Finally, CSF-cNs acquire their typical pear-like shape bearing elongating actin-based protrusions (Fig 1, Stage 3). The same sequence of events was observed in several cells from the 2 subpopulations (ventral and dorso-lateral; Fig 1) and confirmed on fixed tissues after immunostaining at 24, 48 and 72 hpf of LifeAct-GFP-positive CSF-cNs (Fig 2).

Our results indicate that the actin cytoskeleton goes through dynamic and extensive remodeling, suggesting an active participation in CSF-cN morphogenesis. F-actin is first organized at the apical side of the cells to form a ring from which, later on, protrusions seem to arise to form the apical extension.

### The ring of actin colocalizes with CSF-cN apical junctional complexes

The localization and shape of the ring of actin are reminiscent of the circumferential structure formed by actin and adherens and tight junctions at the AJCs in epithelial cells [22]. These complexes are usually lost in newborn neurons to allow their withdrawal from the ventricular zone in order to migrate and differentiate [23,27]. To investigate the localization of AJCs in CSF-cNs relative to the ring of actin, we chose 3 different markers representative of the apical polarization complex—Crumbs 1 (Crb1) [25], adherens junctions—Cadherin 2 (Cdh2) [40], and tight junctions—Zonula-occludens-1 (ZO-1) [41]. At 24 hpf, we observed LifeAct-labeled CSF-cNs displaying the typical ring of actin at their apical side (Fig 2A–2C, arrowheads). At the vicinity of the ring, we also detected Cdh2 (Fig 2A), ZO-1 (Fig 2B), and Crb1 (Fig 2C), confirming that F-actin is enriched at the level of CSF-cN AJCs at 24 hpf.

In 72-hpf larvae, in contrast to ZO-1 being expressed in all cells lining the central canal (Fig 2D and 2F, arrowheads), the expression of Crb1 (Fig 2E and 2F, arrowheads) was specific to CSF-cNs and showed distinct patterns between ventral and dorsolateral subtypes (Fig 2F). In ventral cells, Crb1 formed a narrow subapical ring, as seen in 24-hpf embryos, whereas it expanded in the entire apical extension of dorsolateral cells (Fig 2E and 2F).

These results show that a ring of actin is formed early on in CSF-cNs at the level of the AJCs and that both structures persist after the apical extension is formed. This observation highlights a peculiarity of CSF-cNs compared with other neurons [27] because they retain their apical polarity during differentiation. The initiation and elongation of the actin-based protrusions from the ring of actin formed at the AJCs suggest that the apical complexes participate in the formation of the CSF-cN apical extension.

### Crb1 is required for the morphogenesis of CSF-cN apical extension

**Fig 3:**
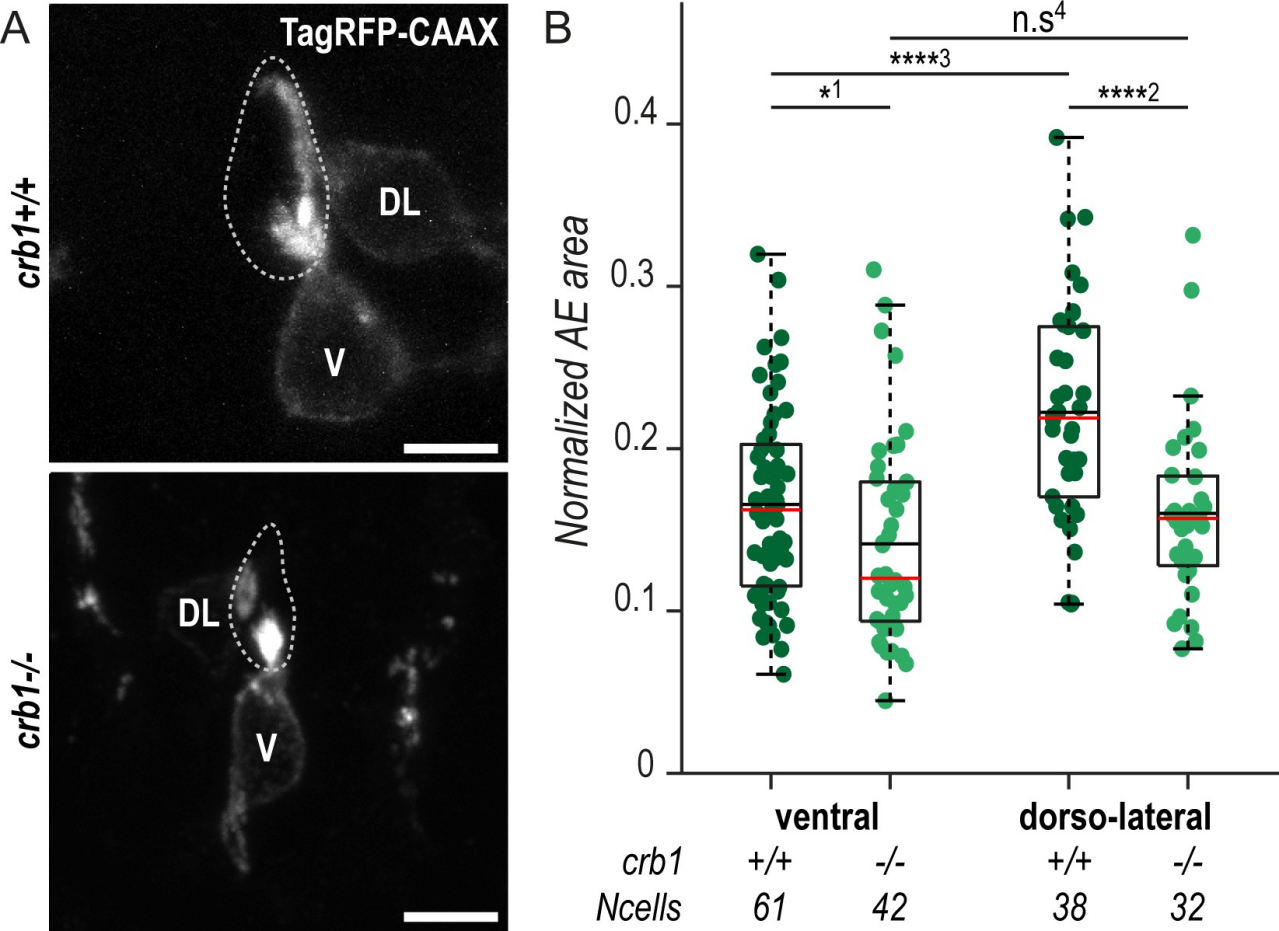
Crb1 participates in the proper development of the CSF-cN apical extension. (A) Z-projections from transversal sections showing V and DL TagRFP-CAAX-expressing CSF-cNs in 72-hpf larvae illustrating the smaller AE in *crb1*^*−/−*^ (bottom panel) compared with wild-type siblings (top panel). The central canal is outlined (dotted lines) according to ZO-1 staining. Scale bars, 5 μm. (B) Quantification of the normalized area covered by CSF-cN AEs at 72 hpf in V and DL cells in *crb1*^*−/−*^ (light green; *N* = 3 fish) compared with wild-type siblings (dark green; *N* = 4 fish). In both CSF-cN subtypes, the AE was significantly smaller in mutant larvae compared with wild-type (*p*_1_ = 0.0477, *p*_2_ = 9.9019 × 10^−5^, *p*_3_ = 2.6381 × 10^−5^, *p*_4_ = 0.1876). Underlying data can be found in S1 Data. AE, apical extension; Crb1, Crumbs 1; CSF-cN, cerebrospinal fluid-contacting neuron; DL, dorsolateral; hpf, hours post fertilization; n.s., not significant; V, ventral; ZO-1, zonula-occludens-1.

Because its expression was specific to CSF-cNs at 72 hpf and showed different patterns between ventral and dorsolateral cells that exhibit distinct morphologies [21], we reasoned that Crb1 might play a special role in the maturation of the apical extension. We therefore knocked out the *crb1* gene using clustered regularly interspaced short palindromic repeats/CRISPR/Cas9-mediated genome editing (S1 Fig). The newly generated mutated allele *crb1*^*icm31*^, thereafter referred to as *crb1*^*−*^, encodes for a truncated protein lacking most functional domains and resulted in the loss of Crb1 immunoreactivity in homozygous mutant larvae (S1 Fig). Analysis of the morphogenesis of CSF-cNs in *crb1*^*−/−*^ animals revealed that mutant cells established a normal apical polarity and presented apical actin-based protrusions (S1 Fig). This result can be explained by the functional redundancy between members of the *crumbs* family allowing the cells lacking Crb1 function to compensate for cell polarization with other Crb proteins [25]. Nonetheless, we noticed that Crb1 loss of function was associated with specific morphological defects of CSF-cN apical extensions at 72 hpf (Fig 3A). Using a previously published method [21], we measured the area covered by the CSF-cN apical extensions in 72-hpf mutant larvae compared with wild-type siblings. We found that mutant dorsolateral and, to a lesser extent, ventral CSF-cNs formed significantly smaller apical extensions (15% reduction in ventral and 28% reduction in dorso-lateral cells) (Fig 3B). This effect resulted in the disappearance of the difference between ventral versus dorsolateral apical extensions in terms of size (Fig 3B) but not in terms of global shape [21].

This result confirms our hypothesis that, beyond its known function as a polarity factor, Crb1 plays a specific role in CSF-cN morphogenesis, which cannot be compensated by other members of the Crb family.

### CSF-cNs specifically express factors involved in the initiation and lengthening of actin-based membrane protrusions

**Fig 4:**
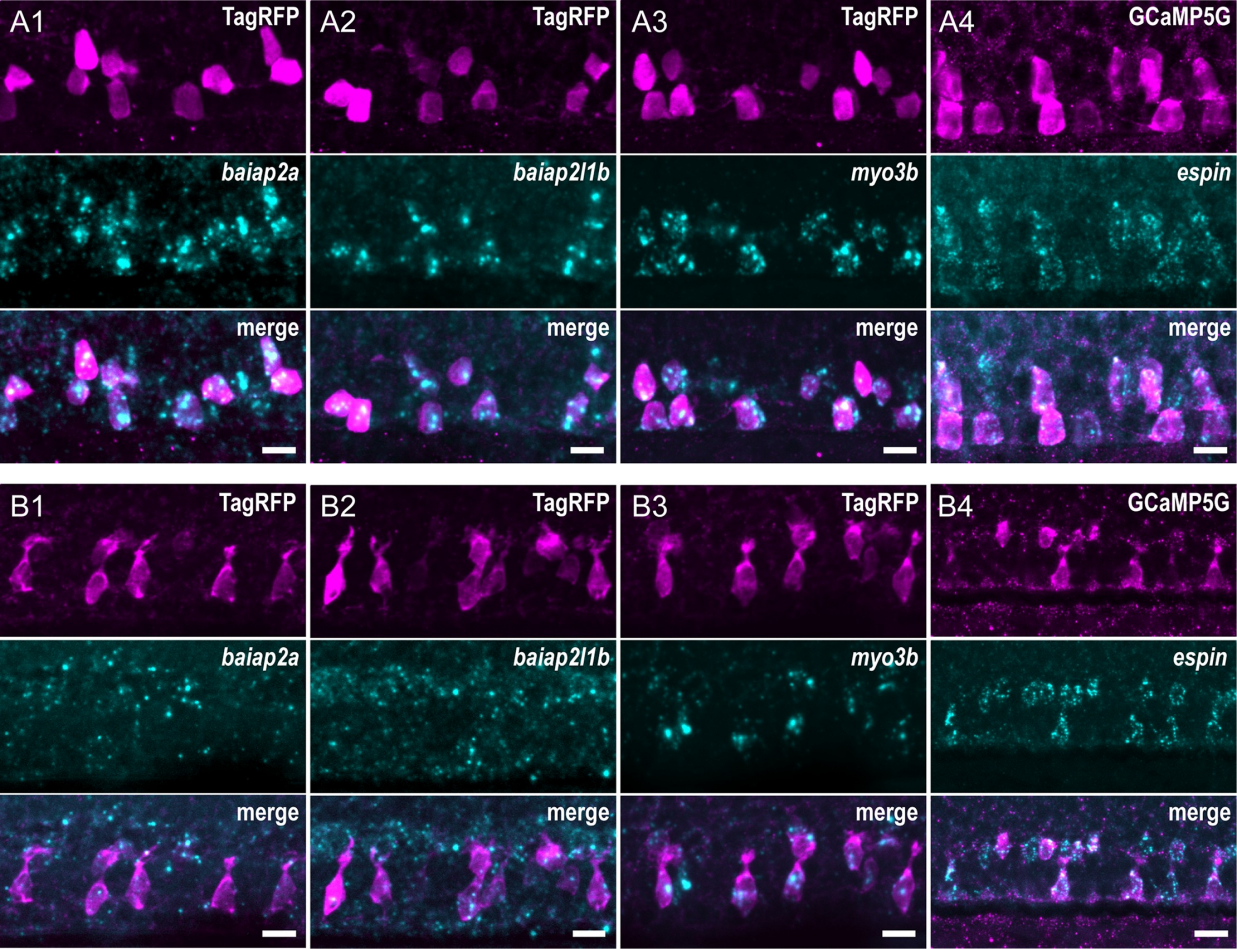
CSF-cNs express a set of known morphogenetic factors. Expression of candidate factors known to be involved in the formation of actin-based protrusions—*baiap2a*, *baiap2l1b*, *myo3b*, and *espin*—assessed by FISH at 24 hpf (A1–4) and 72 hpf (B1–4). Expression in CSF-cNs was validated by combining FISH to IHC for RFP or GFP in *Tg(pkd2l1*:*TagRFP)* (A1–3, B1–3) or *Tg(pkd2l1*:*GCaMP5G)* (A4, B4) transgenic animals, respectively. In 24-hpf embryos, *baipa2a* (A1), *baiap2l1b* (A2), *myo3b* (A3), and *espin* (A4) were enriched in CSF-cNs. In 72-hpf larvae, expression of *baiap2a* (B1) and *baiap2l1b* (B2) was not clearly detected, whereas *myo3b* (B3) and *espin* (B4) remained strongly expressed in CSF-cNs. Scale bars, 10 μm. CSF-cN, cerebrospinal fluid-contacting neuron; FISH, fluorescent in situ hybridization; GFP, green fluorescent protein; hpf, hours post fertilization; IHC, immunohistochemistry; RFP, red fluorescent protein.

We hypothesized that, once formed, the AJC-located actin ring serves as a platform to recruit specific actin-modifying proteins and monomers of actin to enable the initiation and the elaboration of the apical extension. We searched for actin organizers involved in the formation of actin-based protrusions and investigated their expression in CSF-cNs by combining fluorescent in situ hybridization (FISH) with immunohistochemistry (IHC) for fluorescent proteins under the control of the *pkd2l1* promoter to label CSF-cNs. In 24-hpf embryos, we found at least 4 factors specifically enriched in both ventral and dorsolateral CSF-cNs (Fig 4) in the spinal cord: *baiap2a* (Fig 4A1), *baiap2l1b* (Fig 4A2), *myo3b* (Fig 4A3), and *espin* (Fig 4A4). In 72-hpf larvae, *baiap2a* (Fig 4B1) and *baiap2l1b* (Fig 4B2) RNA was not clearly detected in CSF-cNs, whereas both *myo3b* (Fig 4B3) and *espin* (Fig 4B4) still showed strong and specific expression. The patterns of expression of Myo3b and Espin were also confirmed by IHC using specific polyclonal antibodies (S2 Fig) [35,42]. Both proteins are enriched at the apical extension of CSF-cNs in the spinal cord. Furthermore, Espin was clearly detected in various sensory microvilliated cells, namely, olfactory receptor neurons in the olfactory pit and hair cells in the inner ear and lateral line (S2 Fig, panel B).

These observations provided candidate molecular factors potentially involved in the elaboration of the mature CSF-cN apical extension.

### Myo3b is necessary for the localization of Espin and the morphogenesis of the CSF-cN apical extension

**Fig 5:**
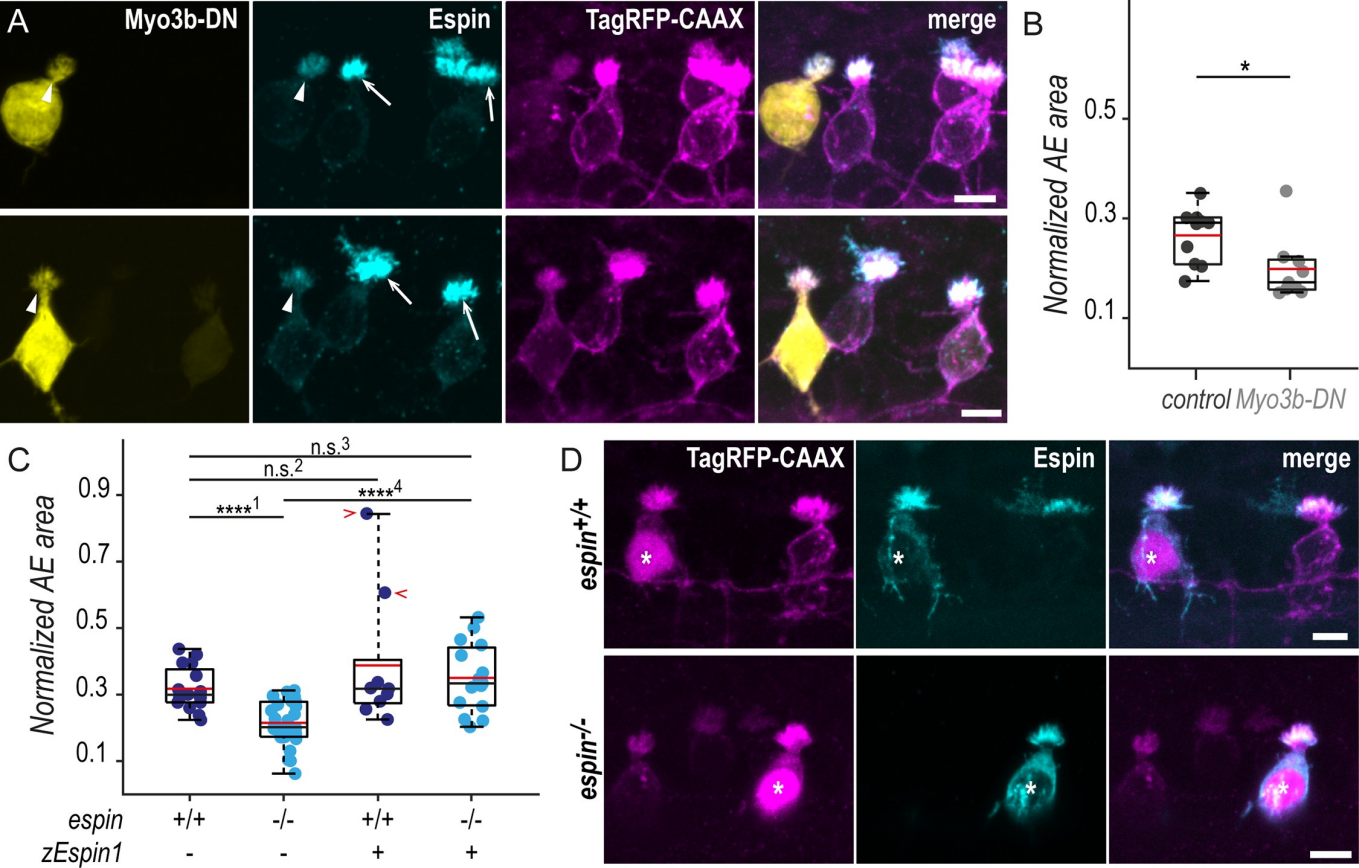
In the absence of Espin actin-bundling activity, CSF-cNs form shorter apical extensions. (A) Z-projections of whole-mounted spinal cords at 72 hpf showing mosaic expression of Myo3b-DN under the control of the *pkd2l1* promoter. Immunostaining reveals Espin (cyan) in TagRFP-CAAX-positive CSF-cNs (magenta) expressing Myo3b-DN (yellow, arrowheads) or not (arrows). In ventral CSF-cNs expressing Myo3b-DN, Espin staining is reduced (observed in 9 cells out of 9), and the AE appears smaller compared with wild-type cells. (B) Quantification of the normalized area covered by the AE of ventral cells expressing Myo3b-DN (*N* = 9 cells) compared with nonexpressing neighboring cells (control*; N* = 10 cells). Cells expressing Myo3b-DN form a significantly smaller AE (*N* = 5 fish; *p* = 0.025). (C) The same quantification was performed in *espin*^*−/−*^ ventral cells at 72 hpf (*N* = 30 cells in 5 fish) compared with wild-type cells (*N* = 16 cells in 5 fish). Mutant cells display significantly smaller AEs (*p*_1_ = 8.4010 × 10^−6^). In wild-type ventral cells, the overexpression of the zEspin1 was sometimes associated with abnormally long microvilli (observed in 2 cells out of 9; red chevrons; *p*_2_ = 0.2123). In *espin*^*−/*−^ cells, the mutant phenotype was rescued by zEspin1 (*N* = 15 cells; *p*_3_ = 0.3029 and *p*_4_ = 3.8556 × 10^−6^). (D) Z-projections of whole-mounted spinal cords at 72 hpf showing mosaic expression of zEspin1 under the control of the *pkd2l1* promoter. Immunostaining for Espin (cyan) in TagRFP-CAAX-positive ventral CSF-cNs (magenta) reveals the loss of Espin immunoreactivity in *espin*^*−/−*^ larvae, which is retrieved in mutant cells expressing zEspin1 (nuclear RFP; magenta; stars). Scale bars, 5 μm. Underlying data can be found in S1 Data. AE, apical extension; CSF-cN, cerebrospinal fluid-contacting neuron; DN, dominant-negative; hpf, hours post fertilization; Myo3b, myosin 3b;n.s., not significant; RFP, red fluorescent protein; zEspin1, zebrafish Espin isoform 1.

The highly specific expression of *myo3b* and *espin* in CSF-cNs, along with their known interaction to promote filopodia lengthening [34,36] and their role in stereocilia morphogenesis in hair cells [34,35,37], motivated us to verify whether these 2 factors contribute to the morphogenesis of CSF-cN apical extensions. We disrupted Myosin 3b (Myo3b) normal function by expressing specifically in CSF-cNs Myo3b-DN, a dominant-negative (DN) form of the protein lacking the motor domain. Mosaic expression of Myo3b-DN enabled us to compare the morphology of cells with reduced Myo3b function with neighboring control ones within the same animal (Fig 5A). At 72 hpf, the expression of Myo3b-DN in CSF-cNs resulted in the reduction of Espin apical staining and of the size of apical extensions (Fig 5A). We quantified the reduction in size by measuring the area covered by apical extensions of ventral CSF-cNs expressing Myo3b-DN or not. We found that cells lacking Myo3b function exhibited significantly smaller apical extensions compared with control cells (25% reduction; Fig 5B).

Our results in CSF-cNs are consistent with previous observations in hair cells and further demonstrate that Myo3b is required for addressing Espin to the actin-based protrusions and is critical for the correct morphogenesis of the apical extension.

### Espin actin-bundling activity is required for the morphogenesis of CSF-cN apical extensions

Espin was shown to rely on its interaction with Myo3b to reach the tip of actin-based protrusions and ensure their lengthening through its actin-bundling activity [34,36]. To test whether the shortening of the apical extension described with Myo3b-DN is due to Espin loss at the CSF-cN apical extension, we generated a mutant allele of *espin* using CRISPR/Cas9-mediated genome editing (S3 Fig). In zebrafish, there is a single *espin* paralog, whom predicted products share a high sequence identity with the main domains described in the rat Espin protein. Because several Espin isoforms have been described in rodents [42–44], we designed our guide RNA against the sequence encoding for the very first amino acids of the conserved actin-bundling module (S3 Fig, panel B). This actin-bundling module is essential for the protein actin-bundling function and present in all described isoforms [42,44,45]. Our newly generated mutant *espin*^*icm26*^ allele, thereafter referred to as *espin*^*−*^, disturbed the sequence of the module from the first amino acid (S3 Fig, panels B-C) and resulted in loss of Espin immunoreactivity at embryonic and larval stages, confirming the efficiency of our knock-out strategy (S3 Fig, panel D). We observed a reduction of Espin staining in heterozygous fish, indicating that the loss of one *espin* wild-type allele is sufficient to reduce protein levels (S3 Fig, panel E).

In 72-hpf *espin*^*−/−*^ larvae, we noticed that CSF-cNs displayed smaller apical extensions than in wild-type siblings (Fig 5D). Using the same analysis than previously for Myo3b-DN, we measured a significant reduction of the area covered by apical extensions in ventral CSF-cNs of homozygous compared with wild-type larvae (32% reduction; Fig 5C). To further ascertain that this reduction in the size of CSF-cN apical extensions was specific to the loss of Espin function, we devised a vector to express the zebrafish Espin isoform 1 (zEspin1) in CSF-cNs under the control of the *pkd2l1* promoter. Mosaic expression of zEspin1 allowed us to compare overexpressing versus wild-type CSF-cNs as well as rescued versus mutant cells within *espin*^*+/+*^ and *espin*^*−/−*^ siblings, respectively (Fig 5D). In 72-hpf wild-type larvae, the overexpression of Espin occasionally led to a drastic increase in apical extension area (Fig 5C, red chevrons), suggesting that Espin level modulates the final size of the apical extension size. In *espin*^*-/-*^ animals, the expression of zEspin1 in a subset of CSF-cNs restored their apical extension size to wild-type values (Fig 5C and 5D).

These results demonstrate that the reduction in the apical extension size observed in our newly generated *espin*^*−/−*^ mutant is specifically due to the loss of Espin in CSF-cNs.

### The actin-bundling factor Espin promotes the elongation of CSF-cN microvilli

**Fig 6:**
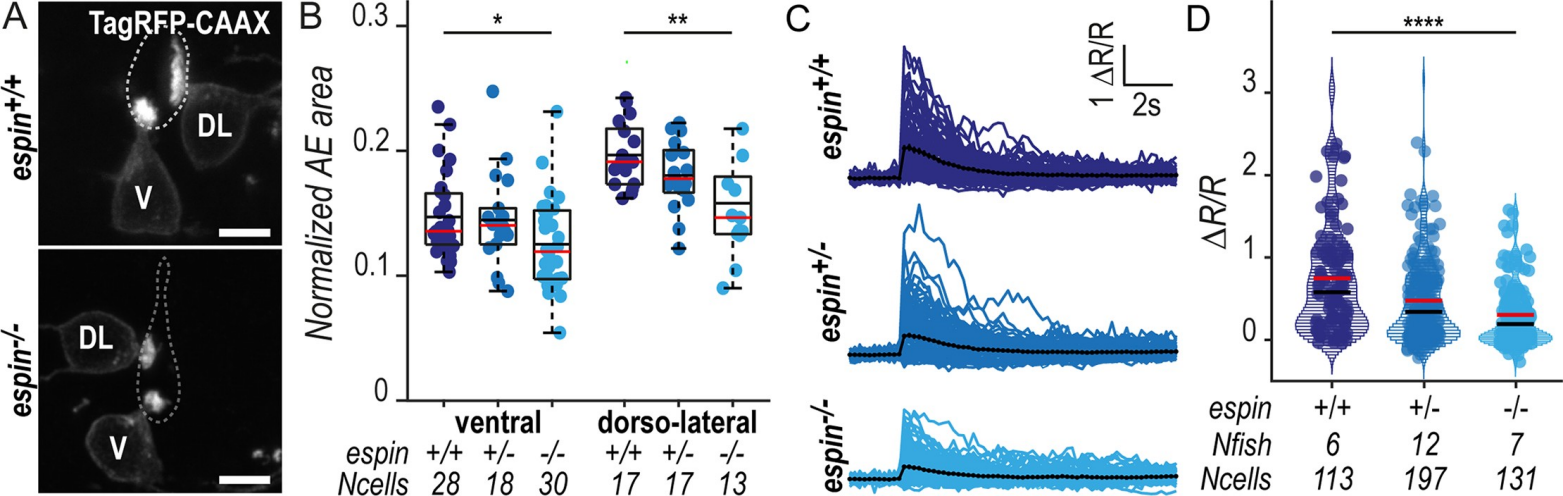
CSF-cNs with shorter apical extensions exhibit reduced mechanosensory response. (A) Z-projections from transversal sections of spinal cords with V and DL TagRFP-CAAX-positive CSF-cNs at 72 hpf illustrating the smaller AEs in *espin*^*−/−*^ (bottom panel) compared with wild-type siblings (top panel). The central canal was outlined (dotted lines) according to ZO-1 staining. Scale bars, 5 μm. (B) Quantification of the normalized area covered by the CSF-cN AE at 72 hpf in V and DL cells in *espin*^*−/−*^ (light blue, *N* = 2 fish), *espin*^*+/−*^ (blue, *N* = 2 fish), and *espin*^*+/+*^ (dark blue, *N* = 3 fish) siblings (1 representative experiment out of 2). In both CSF-cN subtypes, the AE size gradually decreases when cells miss 1 (*espin*^*+/−*^) or 2 (*espin*^*−/−*^) copies of the wild-type allele (*p*_V_ = 0.0112, df = 74, and *t* = 2.3311 in ventral cells; *p*_DL_ = 0.0017, df = 45, and *t* = 3.0935 in DL cells). (C) Overlay of calcium transients in ipsilateral DL CSF-cNs in response to passive tail bending induced by a glass probe in paralyzed control wild-type larvae versus *espin*^*+/−*^ and *espin*^*−/−*^ siblings at 5 days (120 hpf). The average across cells is shown in black (pulled data from 4 experiments). (D) The amplitude of CSF-cN calcium transients shown in (C) is represented as the ratio of peak fluorescence over baseline (ΔR/R) and is gradually reduced in *espin*^*+/−*^ and *espin*^*−/−*^ compared with wild-type siblings in a wild-type allele number-dependent manner (*p* = 1.1102 × 10^−6^, df = 1309, and *t* = 8.462). Underlying data can be found in S1 Data. AE, apical extension; CSF-cN, cerebrospinal fluid-contacting neuron; df, degrees of freedom; DL, dorsolateral; hpf, hours post fertilization; V, ventral; ZO-1, zonula-occludens-1.

To further analyze the impact of Espin loss on the CSF-cN apical extension, we evaluated the precise morphological features of *espin*^*−/−*^ cells on spinal cross sections (Fig 6A). In 72-hpf larvae, we found that both ventral and dorsolateral CSF-cN subtypes form significantly smaller apical extensions following a gradual decrease matching the *espin* gene copy number, with an intermediate phenotype in *espin*^*+/−*^ heterozygotes (Fig 6B). The loss of Espin protein in *espin*^*−/−*^ larvae led to a 15% reduction in apical extension area in ventral CSF-cNs and 20% in dorsolateral cells (Fig 6B). The same effect was observed at 144 hpf (S4 Fig, panel A), demonstrating that the amount of Espin is critical for the morphogenesis of CSF-cN apical extensions.

Because *espin* and *crb1* mutant phenotypes appeared similar, we tested whether Crb1 function is required for Espin localization in CSF-cNs. 72- hpf *crb1*^*−/−*^ larvae showed normal Espin enrichment at the CSF-cN apical extension. Conversely, we found Crb1 protein normally located at AJCs and apical extensions of CSF-cNs in 72-hpf *espin*^*−/−*^ larvae (S5 Fig). We therefore concluded that, although *crb1* and *espin* mutations led to comparable morphological defects, the 2 proteins act through at least partially distinct regulatory pathways to control the morphogenesis of CSF-cN apical extensions.

We then investigated in more details the structure of CSF-cN apical extension in the *espin* mutant using stimulated emission depletion (STED) microscopy to improve imaging resolution compared with standard confocal microscopy (S4 Fig, panel B1). STED microscopy confirmed our previous observation that CSF-cN apical extensions exhibit high density of microvilli and lack of obvious spatial organization [21]. It also enabled us to clearly delineate the region of densely packed membrane—corresponding to actual protrusions—from the base of the apical region—devoid of intense membrane staining—corresponding to the junctional region labeled by ZO-1 (S4 Fig, panel B1, chevrons). The loss of Espin in *espin*^*−/−*^ mutant larvae caused a strong reduction in the height of the membrane-rich protrusions. We therefore measured the vertical extension of the region above ZO-1–labeled junctions (S4 Fig, panel B2) and confirmed that the previously described reduction in the area covered by the apical extension (Figs 5 and 6) is associated with a reduction in length of membrane protrusions in *espin* mutant larvae (36% reduction).

These results demonstrate that Espin actin-bundling activity is necessary for the morphogenesis of CSF-cN apical extensions in a dose-dependent manner, through the regulation of microvilli elongation.

### The size of the CSF-cN apical extension tunes the amplitude of the mechanosensory response

We previously reported that CSF-cNs respond to mechanical stimuli associated with passive and active tail bending in zebrafish larvae [14]. To investigate the functional consequences of the shortening of CSF-cN apical extension, we tested CSF-cN mechanosensory function using the assay previously reported by Böhm and colleagues [14]. In paralyzed wild-type 120-hpf larvae, passive deformation of the tail with a glass probe induced large intracellular calcium transients in ipsilateral dorsolateral CSF-cNs (Fig 6C, S2 Movie). In *espin*^*+/−*^ and *espin*^*−/−*^ larvae, however, CSF-cN calcium transients in response to tail bends were smaller, correlated with wild-type *espin* gene copy number (Fig 6C and 6D and S2 Movie) (38% reduction in *espin*^*+/−*^ and 60% in *espin*^*−/−*^).

Similarly, *crb1*^*−/−*^ CSF-cNs displayed reduced calcium transients compared with cells in wild-type siblings (S6 Fig, panels A and B) (79% reduction of the median).

The CSF-cN mechanoresponse to tail bending was previously found to rely on the activity of the transient receptor potential channel Pkd2l1, enriched at the apical extension [8,13,14]. We therefore checked the localization of the channel in *espin* and *crb1* mutants and found that Pkd2l1 was normally present at the apical extension of CSF-cNs (S6 Fig, panels C and D). It is therefore unlikely that the reduced mechanoresponse observed in mutant cells are explained by defects of Pkd2l1 addressing to the apical extension.

These observations in *crb1* and *espin* mutant larvae demonstrate that the size of CSF-cN apical extension sets the amplitude of the cell mechanosensory response to spine curvature.

## Discussion

### CSF-cNs retain their apical junctional complexes and the accompanying actin ring

To investigate how CSF-cNs develop their apical extension, we undertook a descriptive approach based on long-term time-lapse imaging to monitor F-actin in single cells. We show that early during their differentiation, CSF-cNs display an apical ring of actin beneath the AJCs that they retain throughout differentiation. It is generally thought that newborn cells fated to become neurons withdraw their apical domain—including the primary cilium—while they differentiate [23,27]. Although we cannot exclude an early delamination event followed by the re-establishment of an apical domain before the expression driven by the *pkd2l1* promoter, our results strongly suggest that CSF-cNs are not subject to apical abscission or retraction but rather differentiate while retaining their apical cell polarity and AJCs. This is consistent with previous findings that differentiated CSF-cNs still possess a cilium at their apical extension [5,6,14]. The loss of apical domain has been proposed to allow neuronal differentiation by preventing Sonic Hedgehog (Shh) cilium-dependent signaling [27]. Zebrafish spinal CSF-cNs remain in contact with the source of the Shh ligand, the CSF, throughout differentiation but attenuate their responsiveness to Shh through a decrease in Notch signaling [46]. Whether this constant exposure to the CSF allows the reception of other signals than Shh important for their differentiation remains to be determined.

### Actin-based protrusions originate from the actin ring

Our time-lapse movies suggest that the ring of actin is the site of emergence of actin-based protrusions to form the apical extension. This actin ring could therefore represent a site for the recruitment of the machinery necessary for protrusion initiation, including the IRSp53 family of proteins that we found specifically expressed in CSF-cNs. This idea is further supported by the observation of actin rings at the apical side of the mechanosensory hair cells of the zebrafish lateral line prior to the sensory microvilli differentiation [47]. The initiation of the apical extension directly from the apical actin belt would ensure the correct positioning of the sensory apparatus and its development directly into the extracellular compartment in contact with the medium from which it senses. The establishment of a strong apicobasal polarity and the specific organization of AJCs and F-actin in circumferential belts are landmarks of microvilliated cell types [48]. Therefore, our hypothesis of an involvement of the F-actin circumferential belt in the arising of protrusions offers a new avenue to solve the long-standing problem of apical microvilli initiation in different cell types, like intestinal brush border epithelial cells or microvilliated sensory cells [31,49].

### I-BAR containing factors as initiators of the actin-based protrusions in CSF-cNs

We show that CSF-cNs specifically express transiently at least 2 factors containing an I-BAR domain (for Inverse Bin-Amphiphysin-Rvs167) at 24 hpf—*baiap2a* and *baiap2l1b*. I-BAR domain proteins are capable of binding both actin cytoskeleton and membrane lipids, which enables them to simultaneously sense and induce membrane curvature [50,51]. We know from the literature that I-BAR family members are capable of generating negative membrane curvature and modulating actin dynamics to induce the formation of filopodia [30]. However, the role of these factors in the establishment of more stable microvilli in sensory cell types has never been investigated. Loss of function approaches are challenging in zebrafish due to a high redundancy of IRSp53 factors, and we failed to see an effect with a DN form. To our knowledge, this study is the first evidence for their possible involvement in the initiation of the actin-based protrusions forming a sensory apical extension.

### Special role of Crb1 in CSF-cN apical extension morphogenesis

Our results suggest that polarization factors involved in the establishment of the apical domain and located at the vicinity of AJCs, near the ring of actin, are critical for the formation of the apical extension. One of these factors, Crb1, particularly drew our attention. First, Crb1 is specifically retained in spinal CSF-cNs at 72 hpf, while being down-regulated in the other cells lining the central canal. Second, Crb1 shows distinct patterns of expression in ventral CSF-cNs, where it forms a ring of actin at the vicinity of the AJCs, and dorso-lateral cells, where it covers the entire apical domain and protrusions. Our dominant-negative and knock-out strategies to induce Crb1 loss of function failed to cause early polarization defects in CSF-cNs, a result that can be explained by the high functional redundancy between intracellular domains of the different Crb proteins in zebrafish [52].

However, we find that the spatial restriction of Crb1 to CSF-cN apical domain during differentiation participates in the proper morphogenesis of apical extensions. Our results suggest that the differential localization of Crb1 in the 2 CSF-cN subtypes contributes to the observed difference in size between ventral and dorsolateral cells but does not fully explain the difference in terms of shape [21]. Further investigation will be required to address the mechanisms underlying the formation of distinct apical extension morphologies among CSF-cNs.

Crb proteins have been demonstrated to regulate morphogenesis of sensory protrusions in zebrafish and fly photoreceptors [53,54] as well as wing hairs in *Drosophila* [55]. In humans, mutations in the *crb1* gene, mostly in the region encoding the extracellular domain, causes inherited retinal dystrophies [52]. It was shown that the unique *Drosophila* Crb plays a critical role in the morphogenesis of microvilli-based structures in the photoreceptor cells, rhabdomeres [54]. This role involves the extracellular domain of the protein, whose function is less known and is unique to Crb1 in vertebrates [52,53]. Together with these results, our work suggests that Crb1 function in actin-based protrusion formation is conserved in vertebrates and calls for further studies on the role of the unique Crb1 extracellular domain in the morphogenesis of sensory apical extensions.

### The actin-bundling protein Espin and its interactor Myo3b are key players in the formation of CSF-cN actin-based protrusions

We also show that CSF-cNs express *myo3b* and *espin* during their differentiation. Myo3b is a class III unconventional myosin, whereas Espin is an actin-bundling factor. Actin-bundling factors are able to cross-link actin filaments together and form higher-magnitude actin structures, such as microvilli [31,56,57]. In particular, Espin is commonly found in mechano- and chemosensory microvillliated cells in which this factor is a critical actin organizer in the development and maintenance of the sensory apparatus [58,59]. ESPIN was also found to be a hallmark of the CSF-cN population in the mouse central nervous system in a large-scale single-cell sequencing study [60]. Previous work demonstrated that Espin and Myo3b are interdependent to be transported to the tip of actin-based protrusions, where Espin ensures the lengthening of actin filaments through its actin-bundling activity [34,36]. Our results indicate that CSF-cNs use Myo3b and Espin in a similar fashion to ensure the lengthening of actin-based protrusions forming the apical extension.

Our work further shows that suppressing one actin-bundling factor, Espin, shortens but does not suppress the actin-based CSF-cN apical extension. It has been shown that the development of bundles of actin filaments involves a combination of at least 2 different factors, usually in a sequential manner [29]. The activity of several actin cross-linkers is required to subsequently initiate loose bundling and then tightly pack the bundles into an organized functional structure [61]. Therefore, our work suggests that other actin-bundling factors, yet to be identified, are necessary during the earlier phases of microvilli initiation and elongation of the CSF-cN apical extension.

It is interesting to note here that Baiap factors contain an SRC Homology 3 (SH3) domain, which was shown to strongly interact with the proline-rich regions present in the longest isoforms of Espin [43]. Our results indicate that members of the Baiap family could potentially act together with Espin and Myo3b to coordinate the initiation and the elongation of actin-based protrusions in CSF-cNs.

We describe comparable morphological defects resulting from both *crb1* and *espin* mutations, namely, a reduction of the apical extension size in mutant cells. Because we observe no mislocalization of Espin in *crb1* mutant cells and, conversely, no mislocalization of Crb1 in *espin* mutant cells, it is unlikely that the 2 proteins regulate CSF-cN apical extension morphogenesis through the same pathway. Consequently, the correct morphogenesis of the CSF-cN apical extension appears to rely on at least 2 distinct mechanisms, whose interactions remain unclear. Further investigation of the putative interplay between Crb1, Espin, and their respective interactors, as well as how the 2 pathways converge to support the formation of CSF-cN apical extensions, will bring essential elements to decipher molecular mechanisms underlying the morphogenesis of sensory microvilli organelles.

### The length of microvilli tunes mechanosensory response in CSF-cNs

The functional relevance of Espin has been largely documented in hair cells of the inner ear in which Espin loss is associated with incorrect lengthening of stereocilia, which leads to degeneration of hair cells and deafness [37,62,63]. In this study, we show that loss of Espin in CSF-cNs does not induce degeneration of these sensory cells but impacts, in a dose-dependent manner, microvilli length within the apical extension, a result consistent with the current understanding of Espin actin-bundling functions [45].

The reduction of microvilli length without CSF-cN degeneration in *espin* and *crb1* mutants provided a unique opportunity to investigate the contribution of microvilli to the process of sensory transduction. We show that the reduction of microvilli length is associated with a reduction in amplitude of CSF-cN mechanosensory response to passive bending of the spinal cord, indicating that the CSF-cN apical extension acts as a sensory antenna where mechanotransduction occurs. This observation is consistent with previous studies from our group and others demonstrating that CSF-cN-mediated detection of spinal bending [14], CSF flow and pressure applied against the membrane [11,13] requires the Pkd2l1 channel [13,14], which is confined to the apical extension [13].

The fact that the length of microvilli tunes the amplitude of CSF-cN sensory response while cells retain Pkd2l1 expression suggests that the mechanism underlying mechanotransduction in CSF-cNs is constrained by microvilli length. One possible explanation for such an effect could be that the length of microvilli determines the number of Pkd2l1 channels located at the membrane, which sets the amplitude of CSF-cN response to spinal cord bending. Longer microvilli may, furthermore, enable CSF-cNs to sample changes of CSF flow associated with spinal bending over larger volumes in the central canal and thereby amplify the mechanosensory response. Alternatively, proper lengthening of microvilli may enable the establishment of physical links between microvilli within the apical extension allowing for cohesion and/or mechanotransduction gating in a similar manner to hair cells [64,65]. The investigation of dynamic mechanisms underlying CSF-cN mechanotransduction in relation to the shape of microvilli will be the subject of future studies.

In hair cells of the inner ear, a large body of work has demonstrated the critical role of the staircase organization and patterned elongation of stereocilia for hearing [31]. Our results in CSF-cNs, where microvilli show no obvious organization, suggest that the lengthening of actin-based protrusions nonetheless finely modulates sensory function. Further physiological investigation in other microvilliated sensory cells such as vomeronasal olfactory receptor neurons or solitary chemosensory cells, will reveal whether our observation may be generalized to more microvilliated cells.

## Materials and methods

### Ethics statement

Animal handling and protocols were carried out with the validation of the Institut du Cerveau et de la Moelle épinière in agreement with the French National Ethics Committee (Comité National de Réflexion Ethique sur l’Expérimentation Animale, Ce5/2011/056) and European Communities Council Directive (2010/63/EU). Because experimentation on zebrafish larvae prior to 5 days old does not require approval of a protocol by the ethics committee, our project received the approval from the local ICM health and ethics committee.

### Animal care

Zebrafish adults and embryos and/or larvae were reared and maintained in a 14/10-hour light cycle. Embryos and larvae were raised at 28.5°C until the start of experiments conducted at room temperature. When performed below 48 hpf, staging was assessed by counting somites (30 at 24 hpf) according to Kimmel and colleagues [66].

### Generation and use of transgenic lines

Transgenic lines used in this study are listed in Table 1. Transgenic lines generated using the Tol2 system are already described in [21].

**Table 1:** Transgenic lines used in our study.

Allele name	Transgenic	Labeling	Reference
s1020tEt	*Tg(1020*:*Gal4)*	Motorneurons, CSF-cNs	[74]
Zf518Tg	*Tg(cdh2*:*cdh2-RFP*, *crybb1*:*eCFP)*	Cdh2	[40]
icm07	*Tg(pkd2l1*:*GCaMP5G)*	CSF-cNs	[14]
icm10	*Tg(pkd2l1*:*Gal4)*	CSF-cNs	[39]
icm17	*Tg(pkd2l1*:*TagRFP)*	CSF-cNs	[14]
icm22	*Tg(UAS*:*TagRFP-CAAX;cmlc2*:*eGFP)*	Nonapplicable	[21]
icm28	*Tg(UAS*:*LifeAct-GFP;cryaa*:*V)*	Nonapplicable	[21]

**Abbreviations:** Cdh2, Cadherin 2; CSF-cN, cerebrospinal fluid-contacting neuron.

### Generation of mutant lines and genotyping

We used the CRISPR/Cas9-mediated genome editing system to generate our mutant lines as listed in Table 2. crRNAs were designed using the online CRISPOR program (crispor.tefor.net/) [67] and selected to target the sequence of interest containing a restriction site (S1 and S3 Figs, panels A and B; see also Table 2). crRNA and universal 67mer tracrRNA were ordered from Integrated DNA Technologies. crRNA and tracrRNA were annealed in Duplex buffer and complexed with the Cas9 protein (provided by Jean-Paul Concordet lab) prior to injections into 1 cell-stage wild-type eggs. The efficacy of the crRNA was assessed directly after injection at 48 hpf by performing genotyping on usually 8 pools of 5 injected embryos. The genotyping workflow involved (1) isolating genomic DNA by lysing embryos in proteinase K solution (10 mM Tris [pH 8], 2 mM EDTA, 0.2% Triton X-100, 200 μg/mL proteinase K) after euthanasia in 0.2% MS-222 (Sigma-Aldrich), (2) performing PCR to amplify the target region (FastStart mix, Roche) using forward and reverse primers (as described in Table 2), and (3) digesting the PCR product with the corresponding restriction enzyme. In the presence of the icm26 mutation, the loss of the BstXI site on 1 (+/−) or 2 (−/−) alleles results in the conservation of the intact 350-bp PCR band instead of digested 172- and 182-bp bands in the wild type. In the presence of the icm31 mutation, the loss of the RsaI site on 1 (+/−) or 2 (−/−) alleles results in the conservation of the 25- and 325-bp PCR bands instead of 25-, 132-, and 195-bp bands in the wild type. When efficient editing was observed, siblings of the genotyped embryos were raised to sexual maturity. This F0 generation was screened to find actual transmitters of mutations by crossing them to AB wild-type fish and genotyping the offspring at 48 hpf. When mutations were transmitted, siblings of the genotyped offspring were raised to F1, and adults were genotyped: a piece of fin tissue was sectioned under anesthesia (fin clipping) using 0.02% MS-222 (Sigma-Aldrich), and the PCR product was sequenced (GATC technology) using either the forward or the reverse primer described above to determine the type of mutation. For our study, we selected a 5-bp deletion in exon 11 of *espin* resulting in truncated Espin proteins lacking the conserved ABM (mutated allele named *espin*^*icm26*^) and a 10-bp deletion in exon 2 of *crb1*, resulting in a severely truncated Crb1 protein lacking all domains but one EGF-like extracellular (mutated allele named *crb1*^*icm31*^) (S1 and S3 Figs, panels B-C).

**Table 2:** Mutant lines generated for this study.

Allele name	Mutation	Gene	Target sequence (exon)	Restriction enzyme	PCR primers (5ʹ to 3ʹ)
icm26	5-bp deletion	*espin*	CCAGAACAAGACCAGCGTGG (exon 11)	BstXI	fw: CAAAACCCAACGACACCC rev: ACTTCAACTCATGTTTGGCA
icm31	10-bp deletion	*crb1*	GTACGCTCTGCCAACTGTCC (exon 2)	RsaI	fw: TAACCCTCCTGACAGATGCA rev: AAGACCTCAACACTCTGCCT

**Abbreviations:** crb1, Crumbs 1; fw, forward; rev, reverse.

### Time-lapse imaging

To monitor the formation of the CSF-cN apical extension, we performed live imaging of single CSF-cNs labeled with LifeAct-GFP, a marker of F-actin [38], during early development. To obtain mosaic labeling, we injected approximately 1 ng of the DNA construct *UAS*:*LifeAct-GFP;cryaa*:*Venus* [21] with approximately 3.5 ng Tol2 transposase RNA into *Tg(pkd2l1*:*Gal4)* or *Tg(1020*:*Gal4)* eggs at the 1-cell stage. Imaging was initiated as soon as LifeAct-GFP-positive cells were detected in the spinal cord, usually between 20 and 22 hpf. LifeAct-positive embryos were first anaesthetized in 0.02% MS-222 (Sigma-Aldrich), then mounted laterally in 1.5% low-melting agarose in 50-mm glass-bottom dishes (MatTek #P50G-1.5-14-F). Because the imaging took place at early development of the fish, we made sure to set areas to image, containing at least 1 positive cell for LifeAct, large enough to not lose the cell over time because of developmental growth. Z-stacks spanning the entire cell depth were taken every 5 minutes during several consecutive 1-hour-long sessions using Slidebook software (Intelligent Imaging Innovations, Denver, CO). Imaging was performed using Zeiss ×40 or ×63 water-immersion objectives on a custom spinning disk microscope (Intelligent Imaging Innovations, Denver, CO) with a 488-nm laser.

### Fluorescent IHC and imaging

We followed a similar protocol in fluorescent-positive embryos and larvae after screening. First, samples were fixed in 4% PFA at + 4°C for 4 hours, then washed in PBS Tween 0.01%, blocked for 2–5 hours at room temperature in 1% DMSO, 10% NGS, 0.5% Triton X-100 PBS 1×, and incubated with the following primary antibodies: mouse IgG1 anti–ZO-1 (1/200, Invitrogen #339100), chicken anti-GFP (1/500, Abcam #ab13970), mouse IgG1 anti-RFP (1/500, Thermo Fisher #ma5615257) or rabbit anti-RFP (1/500, Life Technologies #R10367), rabbit anti-Crb1 (1/500; kind gift from Wei lab), rabbit anti-Espin (50 ng/μL, kind gift from Bartles lab), and rabbit anti-Myo3b (1/500; kind gift from Petit lab) in 1% DMSO, 1% NGS, 0.5% Triton X-100 PBS 1X at +4°C overnight. After washing 3 times for at least 10 minutes in PBS 0.1X Tween 0.01% (PBST), samples were incubated with the secondary antibody (usually diluted to 1/500) and DAPI (1/4,000) in the dark, overnight, at +4°C. The next day, embryos or larvae were washed again in PBST for at least 3 times for 30 minutes, and proper staining was assessed by checking the pattern of fluorescence of DAPI control staining before proceeding to mounting. Pkd2l1 staining was performed following an adapted protocol using a polyclonal rabbit antibody as described by Sternberg and colleagues [13]. Samples were mounted using a mounting medium (Vectashield Antifade Mounting) and imaged using an Olympus FV1000 confocal microscope equipped with a ×63 oil immersion objective using the 405, 473, 543 or 567, and 633 or 647 nm laser lines.

### Detailed analysis of the CSF-cN apical extension morphology

Confocal images of the CSF-cN apical extension on spinal cord microtome sections were acquired following the procedure presented in our previous work [21]. We used the stable *Tg(UAS*:*TagRFP-CAAX;cmlc2*:*eGFP)* transgenic line crossed to *Tg(pkd2l1*:*Gal4)* to access the CSF-cN apical extension morphology. To assess the area covered by the CSF-cN apical extension in wild-type or *espin* and *crb1* mutant siblings, we drew polygons outlining the apical extension and the soma on Z-projections using the polygon tool in Fiji.sc/ [68] and used the “area” measurement. For each cell, we normalized the obtained area value by soma area.

### FISH and imaging

All the probes were generated by amplifying coding fragments from zebrafish 72-hpf larva total cDNA. The resulting PCR product was extracted from gel using the QIAquick Gel extraction kit (QIAGEN, Germany) and cloned into the pCRII-Blunt-TOPO vector (Life Technologies, Carlsbad, CA). Clones and orientation were verified by sequencing. For *espin*, the selected plasmid was linearized using NotI, and the resulting product was purified and measured for DNA concentration. For *myo3b*, *baipa2a*, and *baiap2l1b*, the product was generated by a second high-fidelity PCR using T7 forward primer, the corresponding reverse primer (Table 3) and Phu polymerase (Phusion High-Fidelity DNA polymerase, New England BioLabs). Digoxigenin-labeled antisense probes were synthesized from these clones using the T7 RNA polymerase with the RNA labeling kit (Sigma-Aldrich). All probes were then purified using the mini Quick Spin RNA columns (Roche, Basel, Switzerland). In the case of *myo3b*, *baipa2a*, and *baiap2l1b*, probes went through an additional step of hydrolysis by adding 40 mM of NaHCO_3_ and 60 mM Na_2_CO_3_ for 10 minutes at 60°C. The reaction was stopped and precipitated by adding 4 volumes of H_2_O, 0.33 volumes of 3 M NaOA, 0.025 volumes of glacial acetic acid, and 11 volumes of cold ethanol. The primers and restriction enzymes used for every probe are listed in Table 3. Whole-mount FISH was performed on 24-hpf embryos and 72-hpf larvae of the transgenic line *Tg(pkd2l1*:*GCaMP5G)* or *Tg(pkd2l1*:*TagRFP)* prior to fluorescent IHC to reveal GFP or RFP, respectively, to specifically label CSF-cNs. For FISH, samples were prepared by fixation in 4% PFA in PBS overnight at +4°C. At 72 hpf, larvae were treated with Proteinase K in PBST for 20 minutes at 100 μg/mL to ensure the penetration of the probes. Probes were diluted to 1/200 for hybridization overnight at +70°C. To reveal the expression of candidate genes, primary antidigoxigenin-POD coupled antibodies (Sigma-Aldrich) were added to 1/300. Staining was realized using either TAMRA (1/100) in *Tg(pkd2l1*:*GCaMP5G)* samples or FITC (1/200) in *Tg(pkd12l1*:*TagRFP)* tyramide-fluorophore solution for 30 minutes. Imaging was performed on an Olympus FV1000 confocal microscope equipped with a ×20 water immersion objective.

**Table 3:** Primers and restriction enzymes used to generate the FISH probes in this study.

Gene	Primers (from 5ʹ to 3ʹ)	Product size (bp)	Restriction enzyme	Riboprobe hydrolysis?
*espin*	fw: AAGATCTACCGCTTCCTCCACC rev: CCAGCTTGGTTTCATCATAGCCT	825	NotI	No
*myo3b*	fw: TGTTTGATAAAGGATTTTGAGACTCGTC rev: ATATTATATGTACAGCACATTCCGTAGC	3,155	No (use rev primer)	Yes
*baiap2a*	fw: GATAACAAACCACGAGCAGCAAAACAC rev: AGATAATACATGAGGGATTCACAAAACG	2,265	No (use rev primer)	Yes
*baipa2l1b*	fw: GAGTTATTGTGGAGTAAATCGACTGAAC rev: ACAACCTTAAAACAAGCTGAATAATCCG	1,937	No (use rev primer)	Yes

**Abbreviations:** FISH, fluorescent in situ hybridization; fw, forward; rev, reverse.

### Morphological analysis of cells lacking Myo3b

To interfere with Myo3b activity, we built a truncated version of the protein devoid of the motor domain, reasoning that it would act as a dominant-negative form by titrating the interactors of the functional Myo3b [34]. The part of the cDNA encoding the 235 C-terminal amino acids was amplified by PCR (Phusion High-Fidelity DNA polymerase, New England BioLabs) from zebrafish 72-hpf larva total cDNA, using primers containing attB sites for Gateway cloning (fw: 5ʹ-GGGGACAAGTTTGTACAAAAAAGCAGGCTGAATTCACCATGAATCTGATGTTGCGGGAAGTGATCG-3ʹ, and rev: 5ʹ-GGGGACCACTTTGTACAAGAAAGCTGGGTCTCCGGATCCTCTGGAAGAGATAGTGGGCGG-3ʹ). The stop codon was removed, and a GSG sequence was added to allow efficient coexpression of T2A-eGFP [69]. The PCR product was then recombined into the pDONR221 and assembled into the final expression vector in a 3-fragment gateway reaction (ThermoFisher Scientific) using p5E-10XUAS, pME-Myo3b-DN-GSG, p3E-T2A-eGFP, and pDest-cryaa:Venus. p3E-T2A-EGFP was subcloned from pME-HA-UPRT-T2A-EGFP (gift of Teresa Nicolson) [70] using *att*-site tagged primers (fw: 5ʹ-GGGGACAGCTTTCTTGTACAAAGTGGGGGTTAACGGCAGTGGAGAGGG-3ʹ, rev: 5ʹ-GGGGACAACTTTGTATAATAAAGTTGGTTACTTGTACAGCTCGTCCATG-3ʹ). The resulting fragment was inserted into pDONR p2rp3 in a BP recombination reaction (Thermo Fisher). The resulting plasmid was injected into 1-cell eggs carrying a combination of *Tg(UAS*:*TagRFP-CAAX;cmlc2*:*eGFP)* and *Tg(pkd2l1*:*Gal4)*. Confocal images of laterally mounted spinal cords were acquired, and we performed a Z-projection of individual ventral CSF-cNs either expressing Myo3b-DN (GFP and RFP positive) or control (RFP-only positive). The area of the apical extension and the cell body was assessed by drawing a polygon around the structure and measuring the area covered using Fiji.ct/ [68] and normalized by soma area.

### Morphological analysis of *espin*^*icm26/icm26*^ mutant cells and rescue of the phenotype

To assess morphological consequences of the loss of function of Espin in *espin*^*icm26/icm26*^ cells and to compare them with previous observations in cells lacking Myo3b function, we proceeded to the same analysis on confocal images of laterally mounted spinal cords at 72 hpf. To further confirm that the loss of Espin function is responsible for the observed phenotype in both conditions (*espin*^*icm26/icm26*^ and Myo3b-DN), we generated a plasmid to rescue the sole expression of Espin. The cDNA of the Espin isoform 1 was amplified by PCR (Phusion High-Fidelity DNA polymerase, New England BioLabs) from zebrafish 72-hpf larva total cDNA using primers containing attB sites for Gateway cloning (forward: 5ʹ-CACCATGGTGGTTGAAAGGACACTTCTCGC-3ʹ and reverse: 5ʹ-TCCGGATCCCTGTTTCGCTATGTCTCC-3ʹ). The stop codon was removed, and a GSG sequence was added to allow efficient coexpression of T2A-RFP-NLS [69]. The resulting PCR product was recombined into the pENTR TOPO (pENTR/D-TOPO Cloning Kit, ThermoFisher Scientific) to generate pME-zEspin1-GSG. The final rescue vector was assembled in a 3-way Gateway reaction (ThermoFisher Scientific) using p5E-10XUAS, pME-zEspin1-GSG, p3E-T2A-tagRFP-NLS, and pDest-cryaa:mCherry. p3E-T2A-TagRFP-NLS was generated by inserting the TagRFP-NLS cDNA into a p3E-T2A plasmid [71] linearized with AvrII (NEBuilder HiFi DNA Assembly Cloning Kit, New England BioLabs). The TagRFP cDNA was amplified from pME-TagRFP-CAAX [72] by PCR using forward 5ʹ-CGAGGAGAATCCTGGCCCACTTGTGTCTAAGGGCGAAGAG-3ʹ and the nuclear targeting sequence NLS-tagged reverse 5ʹ-GACCGAAATTAATTAAAAACTTAGACTTTCCTCTTCTTCTTGGGATTAAGTTTGTGCCCC-3ʹ primers. The resulting rescue plasmid was injected into 1-cell eggs obtained from *Tg(pkd2l1*:*Gal4*,*UAS*:*TagRFP-CAAX;cmlc2*:*eGFP)espin*^*icm26*^ incross allowing the sparse expression of the rescue zEspin1 in *espin*^*+/+*^, *espin*^*+/icm26*^, and *espin*^*icm26/icm26*^ embryos and larvae.

### STED microscopy imaging of the CSF-cN apical extension

To gain in confocal imaging resolution, we undertook STED microscopy imaging followed by deconvolution treatment on spinal cord microtome sections from a combination of *Tg(UAS*:*TagRFP-CAAX;cmlc2*:*eGFP)* and *Tg(pkd2l1*:*Gal4)* transgenic lines carrying the *espin*^*icm26*^ mutation. The original IHC protocol [21] was adapted using the goat antirabbit IgG Alexa Fluor 594 secondary antibody (#A-11012; ThermoFisher) diluted to 1/200 to reveal the membrane-tagged TagRFP labeling in CSF-cNs in a STED-stable manner. The ZO-1 staining to outline the central canal was kept unmodified. Imaging of both ZO-1 and TagRFP-CAAX was carried out on a Leica TCS SP8 STED 3× confocal microscope using a 93× glycerol objective. ZO-1 labeling was excited with a white laser at 488 nm (3.5%) and detected on a HyD detector from 500 to 550 nm. TagRFP-CAAX labeling was excited with a white light laser at 590 nm (8.5%), depleted with a 775 nm depletion laser (80%), and detected on a HyD detector from 600 to 650 nm, gated from 0.6 to 5.37 ns. Images were then processed by deconvolution using Huygens software (SVI, the Netherlands). To assess the vertical extension of CSF-cN apical extensions in *espin* wild-type and mutant fish, we drew polygons outlining the apical extension and fitted an ellipse to extract the “minor” measurement using the polygon tool in Fiji.sc/ [68].

### Functional analysis of the mutants

To assess the sensory function of the CSF-cNs in mutant versus wild-type siblings, we performed the same passive tail bending assay as described in the work by Böhm and colleagues [14]. *Tg(pkd2l1*:*GCaMP5G*,*pkd2l1*:*TagRFP)espin*^*icm26*^ adult fish were incrossed, and offspring were screened for GCaMP5G and TagRFP fluorescence at 72 hpf after light anesthesia in 0.02% MS-222 (Sigma-Aldrich). At 120 hpf, double positive larvae were embedded in 1.5% low–melting-point agarose and paralyzed by injecting 0.5 nL of α-Bungarotoxin (0.5 mM; Tocris #11032-79-4) in the musculature above the swimming bladder. Half of the tail was freed unilaterally to give access to the glass probe. Calcium imaging was performed using a 2-photon microscope (Intelligent Imaging Innovations) equipped with a ×20 water objective. Both GCaMP and RFP signals were recorded simultaneously at 5 Hz for 770 frames, allowing for 11 probe deflections to bend the tail every 70 frames, starting at 10 frames. Each stimulation therefore lasted 14 seconds. A new version of the Matlab script used in the work by Böhm and colleagues [14] was generated to perform the analysis. Regions of interest were manually selected in Matlab by drawing the CSF-cN cell bodies in the region of deflection. In order to correct for artefactual and/or experimental movements, we registered all image time series based on a TagRFP reference frame built as the average over 50 frames in a quiet period after the first stimulation. Background noise, calculated in a region with no signal, was subtracted to every frame. Raw signal was extracted for both GCaMP and TagRFP channels, and, to correct for motion artefacts, the 3 frames following the stimulation were removed, and the ratio ΔR/R was calculated as (F_GCaMP_(t) × F_0-TagRFP_) ÷ (F_TagRFP_(t) × F_0-GCaMP_) at every time point left with F_0_ the average fluorescence of the 10 frames preceding the stimulation. The calcium transient amplitude was calculated as the difference (ΔR/R)_post_ − (ΔR/R)_pre_, with (ΔR/R)_post_ the average of the 3 frames right after stimulation and (ΔR/R)_pre_ the average of the 10 frames before stimulation.

### Statistics

All statistics were run on Matlab (Mathworks, Massachusetts). In most cases, student *t* tests were used for hypothesis testing. For obviously skewed distributions, we used the two-sample Kolmogorov-Smirnov test. Values are mean (red line) or median (black line). When displayed, the bottom and top edges of box plots indicate the 25th and the 75th percentiles, respectively, and whiskers extend to the most extreme data points not considered as outliers (outliers are plotted individually). To test the impact of wild-type *espin* allele numbers on apical extension size and calcium responses, we used multiple regression. More precisely, we relied on the general linear model (GLM) [73]: the design matrix included 3 regressors of interest (encoding the number of alleles: +/+, +/−, and −/−) and, when applicable, 1 confounding variable (day of the experiment). Statistical significance of the effect of interest (above and beyond confounding factors) was tested using a *t* test. In all figures, * means that p < 0.05, ** means that p < 0.01, *** means that p < 0.001, and **** means that p < 0.0001.

## Supporting information

S1 Data Excel spreadsheet containing, in separate sheets, the underlying numerical data for Figs 3B, 5B, 5C, 6B, 6C, 6D, S4A, S4B2, S6A and S6B. (XLSX)

S1 Movie Time-lapse imaging showing 3 CSF-cNs going from Stage 1 to Stage 3. Typical time-lapse recording used to obtain the results presented in Fig 1. A Z-stack spanning the entire cells was taken every 5 minutes in a live embryo mounted laterally in 1.5% low-melting agarose from 22 hpf and over 8.5 hours. Three cells (1 ventral and 2 dorsolateral cells) were individually marked by LifeAct-GFP under the control of the *pkd2l1* promoter. CSF-cN, cerebrospinal fluid-contacting neuron; hpf, hours post fertilization.
(AVI)

S2 Movie Neuronal activity in response to passive tail bending reported by calcium imaging in 120-hpf (5-day-old) wild-type and *espin^−/−^* mutant larvae. Typical recordings supporting the results in Fig 6C and 6D. TagRFP and GCAMP5G, a green fluorescent calcium indicator, were coexpressed in CSF-cNs under the control of the *pkd2l1* promoter in both wild-type (“+/+,” left panel) or mutant *espin*^*−/−*^ (“icm26/icm26,” right panel) 120-hpf larvae. The 2 signals were recorded at 5 Hz with a 2-photon laser-scanning microscope while the spinal cord of paralyzed animals was deflected with a glass probe. TagRFP signal (upper panels) is used as a reference to correct for motion artefact in all 3 dimensions. GCAMP5G (middle panels) fluorescence varies with calcium concentration and sensory activity. The change of ratio (ΔR/R) between the 2 signals was used to quantify neuronal activity (lower panels, representing traces of different ROIs tracked on the movies above). CSF-cN, cerebrospinal fluid-contacting neuron; hpf, hours post fertilization; RFP, red fluorescent protein; ROI, region of interest.
(AVI)

S1 Fig Crb1 locus organization and generation of the *crb1^icm31^* mutant. (A) Localization and genomic structure of the unique *crb1* locus in zebrafish on Chromosome 22. (B, Top) Genomic region targeted by the sgRNA in exon 2 (sequence in bold), the earliest compatible target region containing a restriction site, here for RsaI, which is lost when editing occurs and enables a 2-step genotyping with a PCR followed by RsaI digestion. (Bottom) Sequence of the *crb1*^*icm31*^ allele generated showing the 10-bp deletion generated by the CRISPR-Cas9 genome editing technique. The early frameshift results in an amino acid sequence disturbed from early on (green) leading to a premature stop codon. (C) Schematics showing the predicted mutant truncated Crb1 protein obtained with the *crb1*^*icm31*^ 10-bp deletion. Green boxes, EGF-like domains; violet boxes, laminin G-like domains. (D) IHC for Crb1 (cyan) showing the loss of immunoreactivity in TagRFP-CAAX-positive CSF-cNs (magenta) in 72-hpf *crb1*^*−/−*^ larvae compared with wild-type siblings. Scale bars, 10 μm. Crb1, Crumbs 1;CSF-cN, cerebrospinal fluid-contacting neuron; EGF, epidermal growth factor; IHC, immunohistochemistry; PAM, protospacer adjacent motif; sgRNA, single guide RNA.
(TIF)

S2 Fig Myo3b and Espin are enriched at the AE of microvilliated sensory cells. (A) IHC for Myo3b shows the enrichment of the protein (cyan) at the level of AEs of TagRFP-CAAX-positive CSF-cNs (magenta) in 72-hpf larvae. Scale bar, 10 μm. (B) IHC for Espin was performed on whole-mount zebrafish 72-hpf larvae. Scale bar, 100 μm. Espin is enriched at the apical extension of various microvilliated sensory cell types: olfactory neurons in the olfactory pit (B1), hair cells of the inner ear (B2), lateral line hair cells (B3), and CSF-cNs (B4). Scale bars, 10 μm. AE, apical extension; CSF-cN, cerebrospinal fluid-contacting neuron; hpf, hours post fertilization; IHC, immunohistochemistry.
(TIF)

S3 Fig Espin locus organization and generation of the *espin^icm26^* mutant. (A) Localization and genomic structure of the unique *espin* locus in zebrafish on Chromosome 8. The conserved actin-bundling module is encoded by exons 11 to 13. (B, Top) Genomic region targeted by the sgRNA in exon 11 (sequence in bold), right upstream of the coding sequence for the actin-bundling module (amino acid sequence indicated in blue). The target region contains a BstXI digestion site, upstream of the PAM, which is disabled when editing occurs. (Bottom) Sequence of the *espin*^*icm26*^ allele generated showing the 5-bp deletion generated by the CRISPR-Cas9 genome editing technique. In *espin*^*icm26*^, the coded amino acid sequence of the actin-bundling module is disturbed from the first codon (green). (C) Schematics showing the predicted mutant truncated Espin protein obtained with the *espin*^*icm26*^ 5-bp deletion. The actin-bundling module is entirely disabled (white box). Green boxes, ankyrin-like repeats; violet boxes, proline-rich regions; red box, WH2 domain; blue box, actin-bundling module. (D) IHC for Espin (cyan) showing the loss of immunoreactivity in TagRFP-CAAX-positive CSF-cNs (magenta) in 72-hpf *espin*^*−/−*^ larvae compared with wild-type siblings. (E) IHC for Espin showing the gradual loss of immunoreactivity in CSF-cNs of *espin*^*+/−*^ *and espin*^*−/−*^ compared with *espin*^*+/+*^ 72-hpf larvae. Samples were analyzed simultaneously, and images were acquired and treated with the same parameters. Scale bars, 10 μm. CSF-cN, cerebrospinal fluid-contacting neuron; hpf, hours post fertilization; IHC, immunohistochemistry; PAM, protospacer adjacent motif; sgRNA, single guide RNA; WH2, WASP (for Wiskott-Aldrich Syndrom protein) homology 2.
(TIF)

S4 Fig Espin is required for the proper lengthening of CSF-cN microvilli. (A) Quantification of the area covered by the CSF-cN apical extension at 144 hpf (6 days) in ventral and dorsolateral cells in *espin*^*−/−*^ mutant larvae (light blue; *N* = 8 fish) compared with wild-type siblings (dark blue; *N* = 4 fish). Both CSF-cN subtypes lacking Espin show a significant reduction of the area covered by their apical extension as observed at 72 hpf (*p*_ventral_ = 0.0019 and *p*_dorso-lateral_ = 0.0164). (B1) STED confocal images from spinal cross sections of 72-hpf *espin*^*+/+*^ or *espin*^*−/*−^ larvae showing apical extensions of ventral TagRFP-CAAX-positive CSF-cNs. The junctional region is highlighted by ZO-1 staining (green). Scale bars, 1 μm. (B2) Quantification of the vertical extension of ventral CSF-cN apical extensions at 72 hpf in *espin*^*−/*−^ mutant larvae (light blue) versus wild-type larvae (dark blue) from STED images obtained as in (B1). Mutant cells formed significantly shorter apical extensions (*p* = 2.5571 × 10^−4^), suggesting the critical role of Espin actin-bundling activity for the proper lengthening of CSF-cN microvilli. Underlying data can be found in S1 Data. AE, apical extension; CSF-cN, cerebrospinal fluid-contacting neuron; hpf, hours post fertilization; STED, stimulated emission depletion; ZO-1, zonula-occludens-1.
(TIF)

S5 Fig *espin* and *crb1* mutant CSF-cNs retain the expression of Crb1 and Espin proteins, respectively. (A) IHC for Crb1 (cyan) in TagRFP-CAAX-positive CSF-cNs (magenta) of 72-hpf larvae shows that Crb1 is similarly expressed and located in mutant CSF-cNs compared with wild-type cells. (B) IHC for Espin (cyan) and ZO-1 (magenta) to highlight the junctional region in *crb1*^*−/*−^ larvae shows that Espin is similarly expressed and enriched at the apical extension of mutant V and DL CSF-cNs compared with wild-type cells. Scale bars, 10 μm. Crb1, Crumbs 1; CSF-cN, cerebrospinal fluid-contacting neuron; DL, dorso-lateral; hpf, hours post fertilization; IHC, immunohistochemistry; V, ventral; ZO-1, zonula-occludens-1.
(TIF)

S6 Fig CSF-cNs with shorter apical extensions exhibit reduced mechanoresponse in a Pkd2l1-independent manner. (A) Overlay of calcium transients in ipsilateral dorsolateral CSF-cNs in response to tail bending induced by a glass probe in paralyzed wild-type versus *crb1*^*−/−*^ 120-hpf animals (data pooled from 3 experiments). (B) The amplitude of CSF-cN calcium transients shown in (A) is represented as the ratio of peak fluorescence over baseline (ΔR/R) and is significantly reduced in crb1^*−/−*^ mutant compared with wild-type siblings (*p* = 1.012 × 10^−5^). (C and D) IHC for Pkd2l1 channel (cyan) in *espin*^*−/*−^ (C) or *crb1*^*−/−*^ (D) shows that TagRFP-CAAX-positive CSF-cNs (magenta) retain the expression of the channel at their apical extension in mutant larvae similarly to wild-type siblings. Scale bars, 10 μm. Underlying data can be found in S1 Data. AE, apical extension; CSF-cN, cerebrospinal fluid-contacting neuron; hpf, hours post fertilization; IHC, immunohistochemistry.
(TIF)

## Abbreviations

AJC: apical junctional complex
ASIC: Acid-Sensing Ion Channels
Baiap: Brain-specific angiogenesis inhibitor 1-associated protein
Cdh2: Cadherin 2
Crb1: Crumbs 1
CRISPR/Cas9: Clustered Regularly Interspaced Short Palindromic Repeats/CRISPR-Associated Protein 9
CSF-cN: cerebrospinal fluid-contacting neuron
DN: dominant-negative
FISH: fluorescent in situ hybridization
GFP: green fluorescent protein
GLM: general linear model
gRNA: guide RNA
hpf: hours post fertilization
I-BAR: Inverse Bin-Amphiphysin-Rvs167
IHC: immunohistochemistry
IRSp53: Insulin Receptor Substrate protein of 53 kDa
Myo3b: Myosin 3b
Pkd2l1: Polycystic kidney disease-2-like 1
RFP: red fluorescent protein
Shh: Sonic hedgehog
SH3: SRC Homology 3
STED: stimulated emission depletion
zEspin1: zebrafish Espin isoform 1
ZO-1: Zonula Occludens-1
