# Supplementary figures and images for: Regulation of the apical extension morphogenesis tunes the mechanosensory response of microvilliated neurons
Source: PLoS Biol. 2019 Apr 19;17(4):e3000235. doi: 10.1371/journal.pbio.3000235 (PMC6493769; doi:10.1371/journal.pbio.3000235)

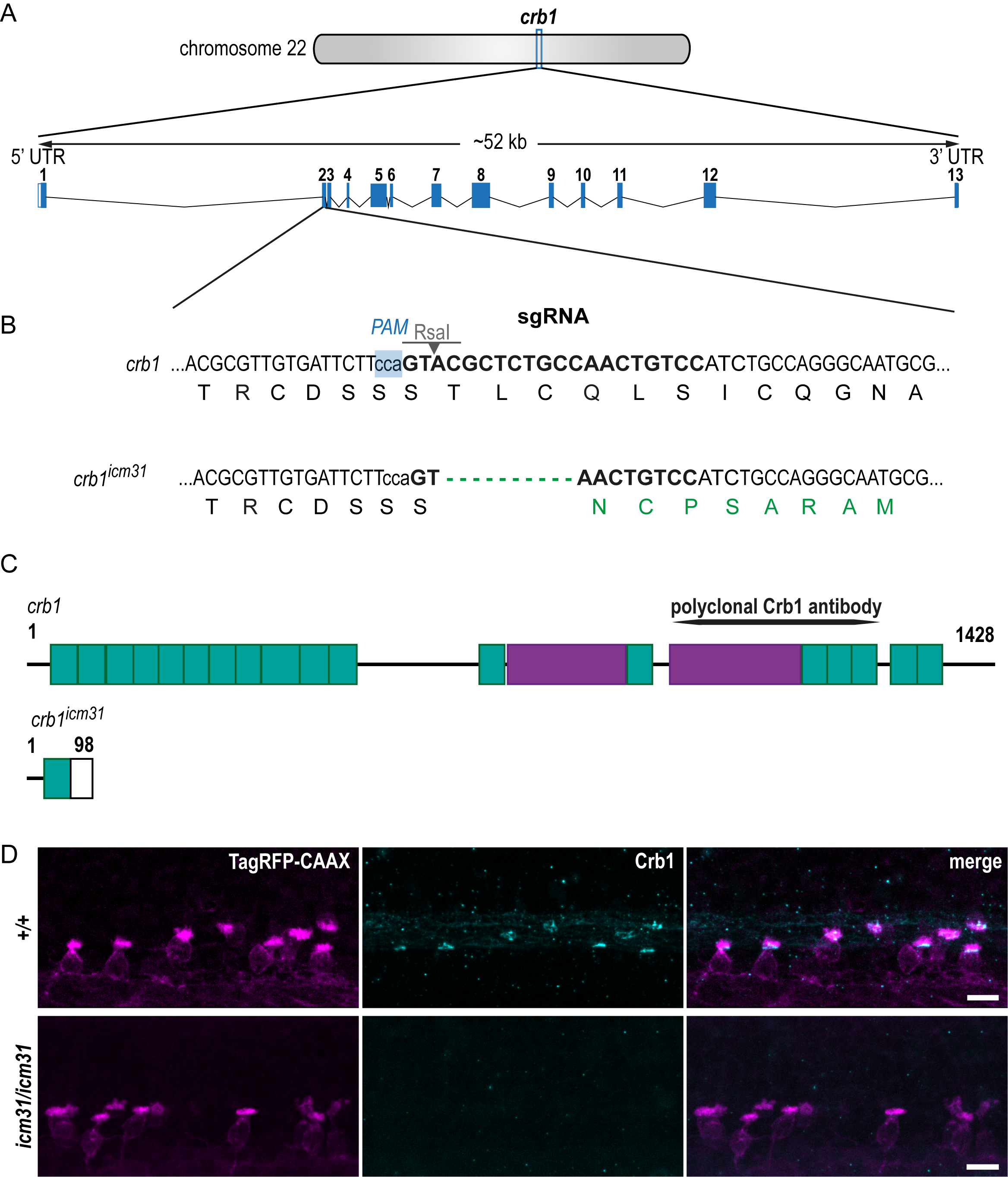

Supplement: S1 Fig — (A) Localization and genomic structure of the unique crb1 locus in zebrafish on Chromosome 22. (B, Top) Genomic region targeted by the sgRNA in exon 2 (sequence in bold), the earliest compatible target region containing a restriction site, here for RsaI, which is lost when editing occurs and enables a 2-step genotyping with a PCR followed by RsaI digestion. (Bottom) Sequence of the crb1icm31 allele generated showing the 10-bp deletion generated by the CRISPR-Cas9 genome editing technique. The early frameshift results in an amino acid sequence disturbed from early on (green) leading to a premature stop codon. (C) Schematics showing the predicted mutant truncated Crb1 protein obtained with the crb1icm31 10-bp deletion. Green boxes, EGF-like domains; violet boxes, laminin G-like domains. (D) IHC for Crb1 (cyan) showing the loss of immunoreactivity in TagRFP-CAAX-positive CSF-cNs (magenta) in 72-hpf crb1−/− larvae compared with wild-type siblings. Scale bars, 10 μm. Crb1, Crumbs 1;CSF-cN, cerebrospinal fluid-contacting neuron; EGF, epidermal growth factor; IHC, immunohistochemistry; PAM, protospacer adjacent motif; sgRNA, single guide RNA. (TIF) [file pbio.3000235.s004.tif]

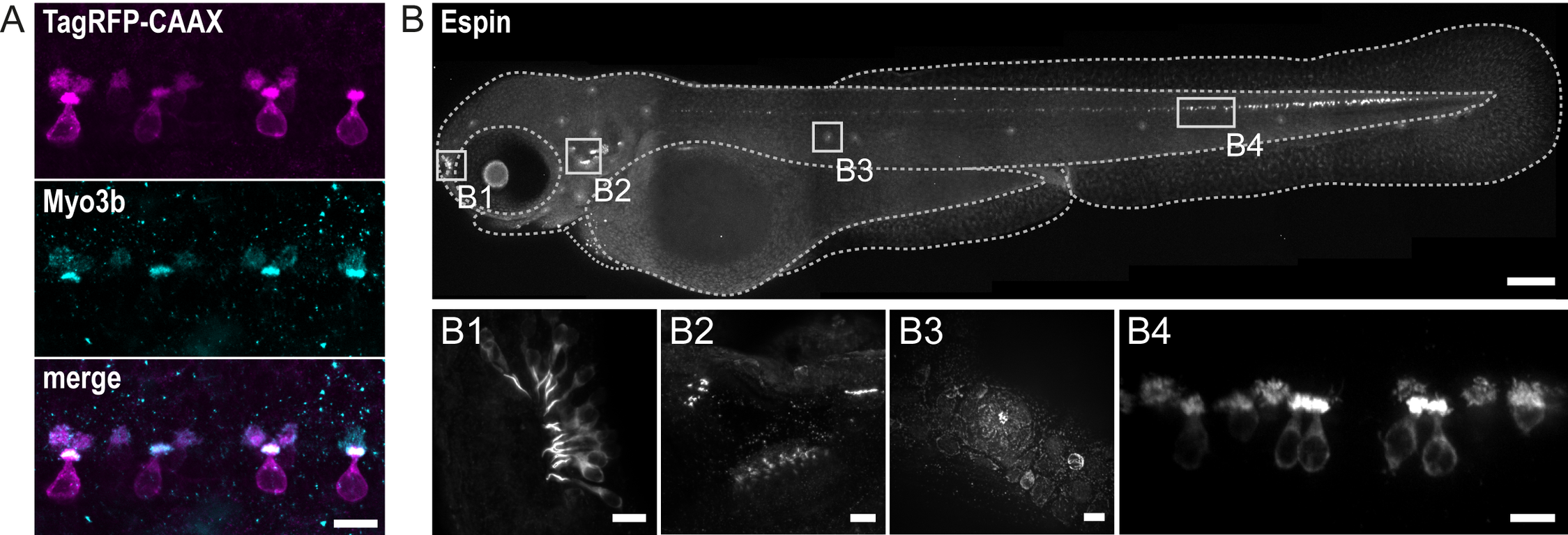

Supplement: S2 Fig — (A) IHC for Myo3b shows the enrichment of the protein (cyan) at the level of AEs of TagRFP-CAAX-positive CSF-cNs (magenta) in 72-hpf larvae. Scale bar, 10 μm. (B) IHC for Espin was performed on whole-mount zebrafish 72-hpf larvae. Scale bar, 100 μm. Espin is enriched at the apical extension of various microvilliated sensory cell types: olfactory neurons in the olfactory pit (B1), hair cells of the inner ear (B2), lateral line hair cells (B3), and CSF-cNs (B4). Scale bars, 10 μm. AE, apical extension; CSF-cN, cerebrospinal fluid-contacting neuron; hpf, hours post fertilization; IHC, immunohistochemistry. (TIF) [file pbio.3000235.s005.tif]

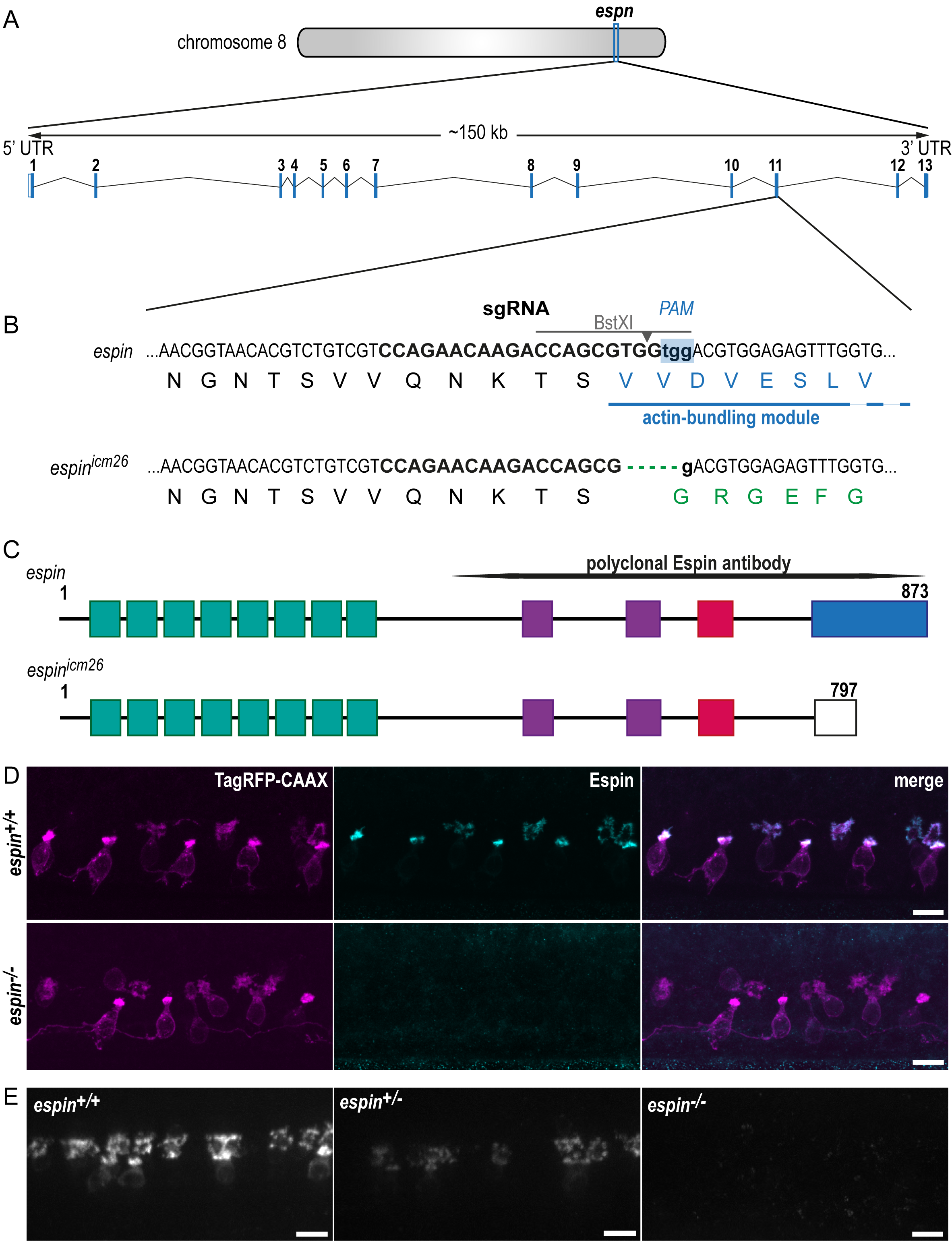

Supplement: S3 Fig — (A) Localization and genomic structure of the unique espin locus in zebrafish on Chromosome 8. The conserved actin-bundling module is encoded by exons 11 to 13. (B, Top) Genomic region targeted by the sgRNA in exon 11 (sequence in bold), right upstream of the coding sequence for the actin-bundling module (amino acid sequence indicated in blue). The target region contains a BstXI digestion site, upstream of the PAM, which is disabled when editing occurs. (Bottom) Sequence of the espinicm26 allele generated showing the 5-bp deletion generated by the CRISPR-Cas9 genome editing technique. In espinicm26, the coded amino acid sequence of the actin-bundling module is disturbed from the first codon (green). (C) Schematics showing the predicted mutant truncated Espin protein obtained with the espinicm26 5-bp deletion. The actin-bundling module is entirely disabled (white box). Green boxes, ankyrin-like repeats; violet boxes, proline-rich regions; red box, WH2 domain; blue box, actin-bundling module. (D) IHC for Espin (cyan) showing the loss of immunoreactivity in TagRFP-CAAX-positive CSF-cNs (magenta) in 72-hpf espin−/− larvae compared with wild-type siblings. (E) IHC for Espin showing the gradual loss of immunoreactivity in CSF-cNs of espin+/− and espin−/− compared with espin+/+ 72-hpf larvae. Samples were analyzed simultaneously, and images were acquired and treated with the same parameters. Scale bars, 10 μm. CSF-cN, cerebrospinal fluid-contacting neuron; hpf, hours post fertilization; IHC, immunohistochemistry; PAM, protospacer adjacent motif; sgRNA, single guide RNA; WH2, WASP (for Wiskott-Aldrich Syndrom protein) homology 2. (TIF) [file pbio.3000235.s006.tif]

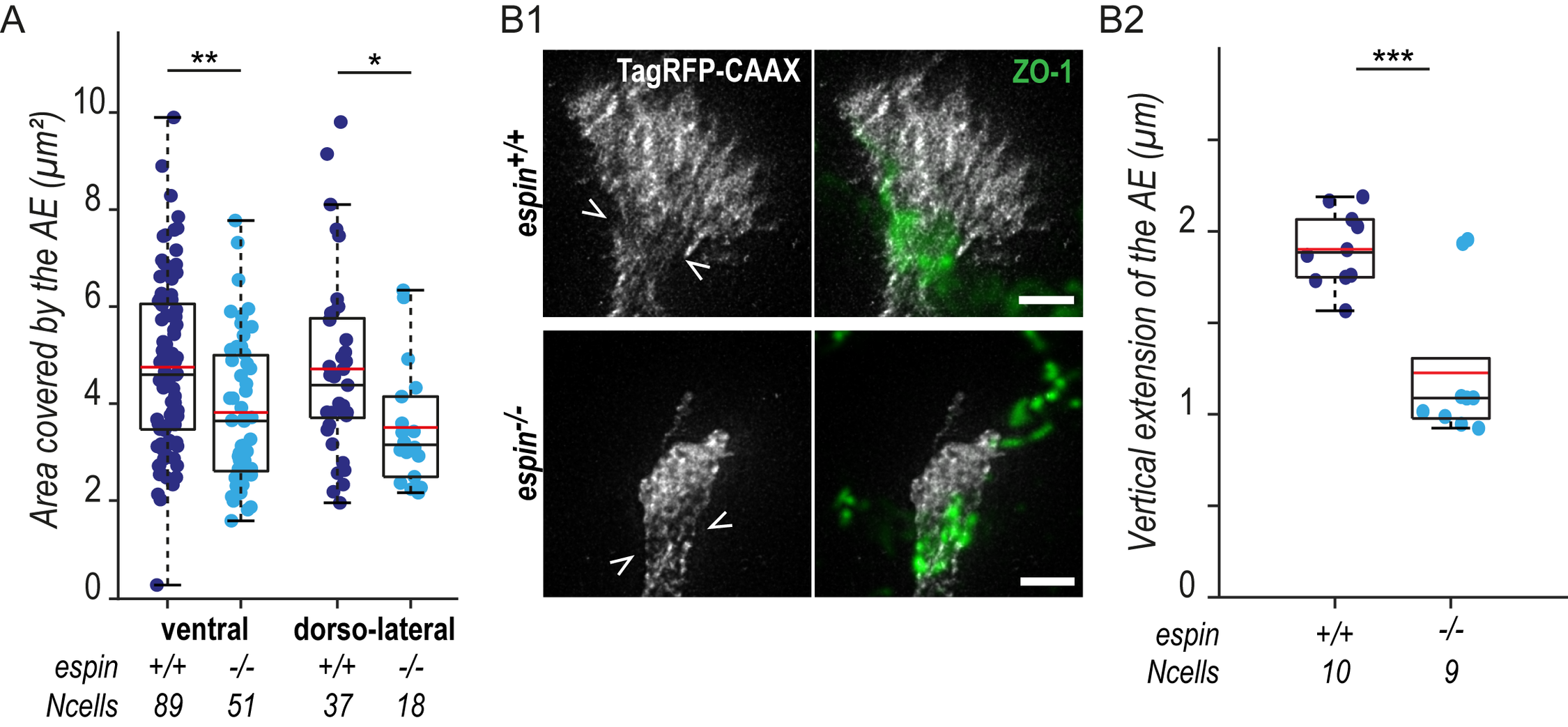

Supplement: S4 Fig — (A) Quantification of the area covered by the CSF-cN apical extension at 144 hpf (6 days) in ventral and dorsolateral cells in espin−/− mutant larvae (light blue; N = 8 fish) compared with wild-type siblings (dark blue; N = 4 fish). Both CSF-cN subtypes lacking Espin show a significant reduction of the area covered by their apical extension as observed at 72 hpf (pventral = 0.0019 and pdorso-lateral = 0.0164). (B1) STED confocal images from spinal cross sections of 72-hpf espin+/+ or espin−/− larvae showing apical extensions of ventral TagRFP-CAAX-positive CSF-cNs. The junctional region is highlighted by ZO-1 staining (green). Scale bars, 1 μm. (B2) Quantification of the vertical extension of ventral CSF-cN apical extensions at 72 hpf in espin−/− mutant larvae (light blue) versus wild-type larvae (dark blue) from STED images obtained as in (B1). Mutant cells formed significantly shorter apical extensions (p = 2.5571 × 10^−4^), suggesting the critical role of Espin actin-bundling activity for the proper lengthening of CSF-cN microvilli. Underlying data can be found in S1 Data. AE, apical extension; CSF-cN, cerebrospinal fluid-contacting neuron; hpf, hours post fertilization; STED, stimulated emission depletion; ZO-1, zonula-occludens-1. (TIF) [file pbio.3000235.s007.tif]

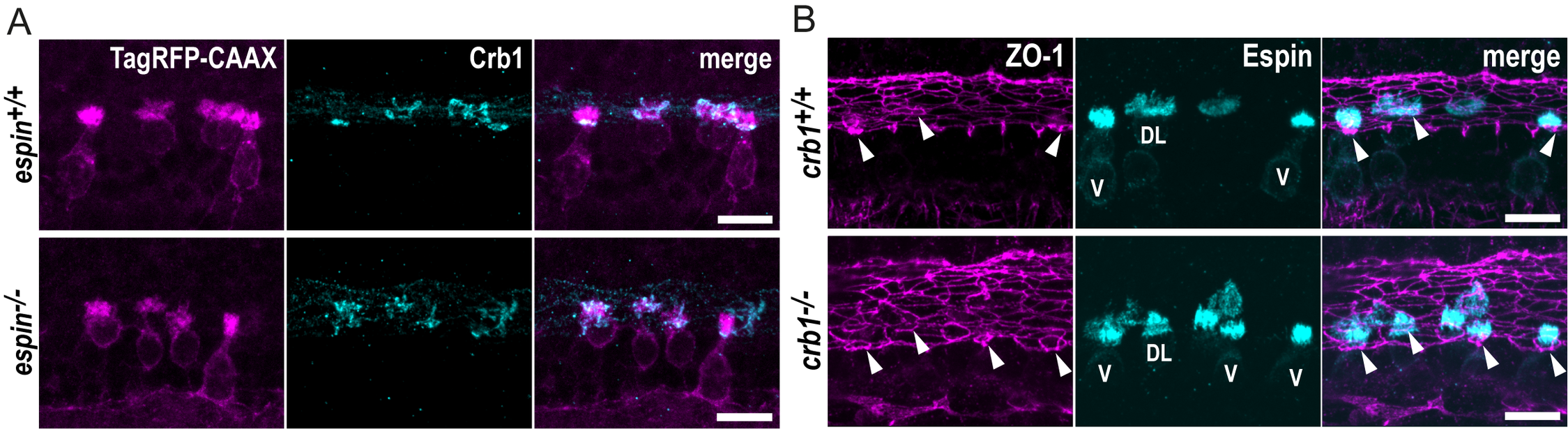

Supplement: S5 Fig — (A) IHC for Crb1 (cyan) in TagRFP-CAAX-positive CSF-cNs (magenta) of 72-hpf larvae shows that Crb1 is similarly expressed and located in mutant CSF-cNs compared with wild-type cells. (B) IHC for Espin (cyan) and ZO-1 (magenta) to highlight the junctional region in crb1−/− larvae shows that Espin is similarly expressed and enriched at the apical extension of mutant V and DL CSF-cNs compared with wild-type cells. Scale bars, 10 μm. Crb1, Crumbs 1; CSF-cN, cerebrospinal fluid-contacting neuron; DL, dorso-lateral; hpf, hours post fertilization; IHC, immunohistochemistry; V, ventral; ZO-1, zonula-occludens-1. (TIF) [file pbio.3000235.s008.tif]

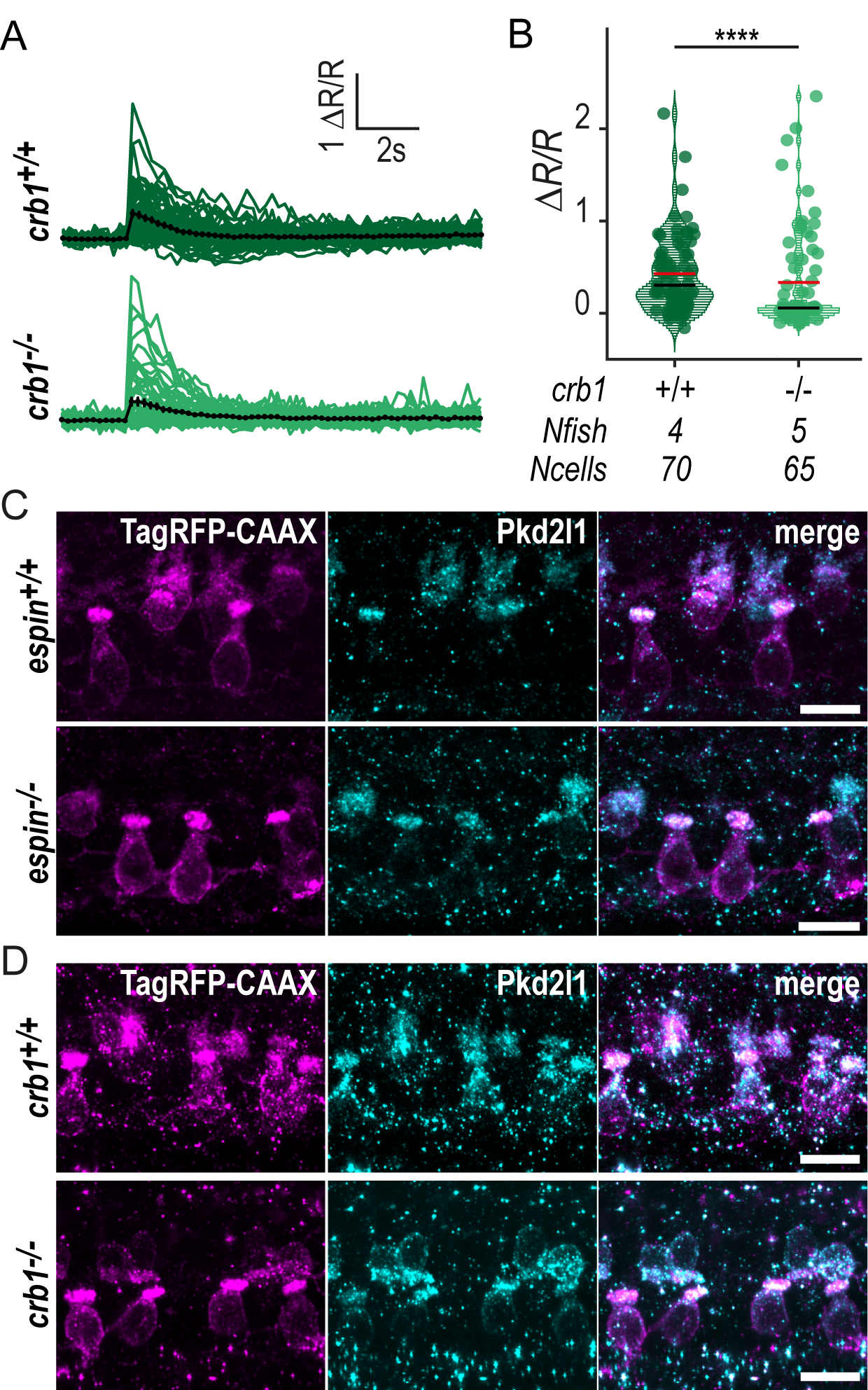

Supplement: S6 Fig — (A) Overlay of calcium transients in ipsilateral dorsolateral CSF-cNs in response to tail bending induced by a glass probe in paralyzed wild-type versus crb1−/− 120-hpf animals (data pooled from 3 experiments). (B) The amplitude of CSF-cN calcium transients shown in (A) is represented as the ratio of peak fluorescence over baseline (ΔR/R) and is significantly reduced in crb1−/− mutant compared with wild-type siblings (p = 1.012 × 10^−5^). (C and D) IHC for Pkd2l1 channel (cyan) in espin−/− (C) or crb1−/− (D) shows that TagRFP-CAAX-positive CSF-cNs (magenta) retain the expression of the channel at their apical extension in mutant larvae similarly to wild-type siblings. Scale bars, 10 μm. Underlying data can be found in S1 Data. AE, apical extension; CSF-cN, cerebrospinal fluid-contacting neuron; hpf, hours post fertilization; IHC, immunohistochemistry. (TIF) [file pbio.3000235.s009.tif]
